# Aberrant CX3CL1-CX3CR1 Signaling Reprograms Microglial Exosome Secretion via KIFC2 to Drive Cognitive Impairment in Chronic Pain

**DOI:** 10.3390/ijms27146304

**Published:** 2026-07-15

**Authors:** Chen Hu, Xinlu Zhang, Wei Zhao, Wenjun Ke, Haoxiang Ma, Wenna Sang, Qian Gao

**Affiliations:** School of Life Science, Anhui Medical University, Hefei 230032, China; 19956040927@163.com (C.H.);

**Keywords:** neuroinflammation, extracellular vesicles, interleukin-17, neurotoxicity, memory deficits

## Abstract

Chronic pain acts as a potent driver of progressive cognitive impairment. Although microglial hyperactivation serves as a pivotal mechanistic bridge in this comorbidity, the intracellular molecular cascades coupling persistent nociception to cognitive decline remain largely elusive. Here, we identify a previously unrecognized microglial secretome remodeling axis, governed by CX3CL1-CX3CR1 signaling, that drives pain-associated cognitive impairment. Clinically, elevated cerebrospinal fluid (CSF) CX3CL1 correlates strongly with cognitive impairment in chronic pain patients. In murine models, pharmacological blockade of the microglial CX3CL1-CX3CR1 signaling attenuated chronic pain-induced memory deficits. Mechanistically, aberrant CX3CL1-CX3CR1 activation triggers a sequential p38 MAPK-NF-κB cascade to upregulate the kinesin motor KIFC2. This KIFC2 surge fundamentally reprograms microglial vesicular trafficking, driving the massive release of IL-17-enriched small exosomes (<100 nm) that subsequently induce synaptic deterioration and neuronal apoptosis manifested by PSD95 degradation, caspase-3 cleavage, and compromised cell viability. Crucially, this microglial p38 MAPK-NF-κB-KIFC2 cascade hyperactivation was validated in situ within the hippocampal slices of chronic pain models. Collectively, our findings delineate a comprehensive cascade spanning from receptor hyperactivation to KIFC2-dependent exosomal remodeling, elucidating a novel mechanism of microglia-mediated neurotoxicity. Targeting this CX3CL1-KIFC2 exosomal axis offers a potential therapeutic strategy to uncouple chronic pain from its debilitating cognitive comorbidities.

## 1. Introduction

Chronic pain, clinically defined as pain persisting for over three months, afflicts approximately 20% of the global adult population and presents a severe socioeconomic burden [1]. Beyond physical discomfort, chronic pain serves as a key driver of progressive cognitive impairment, manifesting as pervasive deficits across attention, memory, executive control, and decision-making [2,3]. Concomitant cognitive decline affects over 50% of chronic pain outpatients [4,5]. The risk of mild cognitive impairment also increases in proportion to pain severity [6]. As a critical transitional phase before dementia, cognitive impairment not only blunts the clinical efficacy of pain management and diminishes patient quality of life, but also accelerates the progression toward neurodegeneration [7]. Specifically, individuals with single-site chronic pain face a 20% elevated risk of subsequent dementia compared to pain-free cohorts, whereas those with multisite pain exhibit a twofold higher risk [8]. Unfortunately, standard analgesics like opioids offer limited efficacy in improving cognitive dysfunction and may paradoxically aggravate memory deficits by disrupting synaptic plasticity [9]. Therefore, understanding the mechanisms behind chronic pain-induced cognitive impairment is urgent for developing effective treatments.

Growing evidence underscores microglial hyperactivation as the pivotal mechanistic bridge linking chronic pain to cognitive decline. As the primary immune cells of the central nervous system (CNS), microglia become pathologically active in the hippocampus after chronic pain input [10,11]. This aberrant state triggers a robust neuroinflammatory cascade characterized by excessive cytokine release, which subsequently reduces synaptic protein levels and disrupts neuroplasticity, resulting in pronounced cognitive deficits [12,13,14]. Crucially, pharmacological suppression of microglial activation can significantly reverse these synaptic pathologies and improve cognitive decline [12,13,15]. A recent landmark study demonstrated that abnormal hippocampal remodeling in chronic pain is fundamentally driven by dysregulated newborn neuron-microglia crosstalk; whereas neuronal silencing alleviates pain-associated affective pathologies to the detriment of cognitive function, targeted microglial modulation selectively uncouples affective deficits without cognitive cost [16]. A key mechanism underlying such neuron-microglia communication is the CX3CL1 (fractalkine) signaling axis, comprising the unique neuron-derived chemokine CX3CL1 and its cognate microglial receptor, CX3CR1 [17]. Under physiological conditions, CX3CL1-CX3CR1 signaling inhibits microglial activation, reduces unnecessary inflammation, and promotes neuronal survival [17]. However, under pathological conditions such as sustained inflammatory stress or aging, this axis inverts its function, driving prolonged microglial activation, increasing neurotoxic cytokine production, and compounding neuronal damage [18,19]. While CX3CL1-CX3CR1 signaling has been clearly linked to pain development [20], its downstream molecular mechanisms in driving chronic pain-induced cognitive impairment remain elusive.

A key pathway for this profound microglia-neuron crosstalk is the secretion of extracellular vesicles (EVs). Primarily classified by biogenesis and size, these heterogeneous lipid-enclosed particles comprise plasma membrane-derived microvesicles (>200 nm) and endolysosomal-derived exosomes (50–150 nm), the latter secreted via multivesicular body (MVB) fusion with the cell surface [21]. Under physiological conditions, resting microglia secrete a baseline profile of these EVs to maintain CNS homeostasis. However, pathological stressors can trigger a profound structural and molecular reprogramming of the microglial secretome. Accumulating evidence demonstrates that hyperactivated microglia undergo a major shift in secretory dynamics under diverse neuropathological conditions such as environmental toxicity [22], metabolic dysfunction [23], and traumatic brain injury [24], thereby driving the release of pathogenic EVs. After these disease-associated small EVs are taken up by target neurons, they deliver harmful cargo, including neurotoxic microRNAs and pro-inflammatory cytokines, which precipitate synaptic structural damage, aberrant protein phosphorylation, neuronal death, and subsequent cognitive deficits [22,25,26]. Conversely, targeted modulation of microglial activation can reverse this secretory profile towards a neuroprotective phenotype, effectively protecting synaptic plasticity [24,27]. Despite the recognized importance of microglial EVs in mediating neurodegeneration across diverse CNS disorders, their specific role in chronic pain-induced cognitive impairment remains unexplored.

To address this gap, we investigated the causal role and downstream molecular mechanisms of microglial CX3CL1-CX3CR1 signaling in chronic pain-induced cognitive impairment. Leveraging the clinical finding that elevated cerebrospinal fluid (CSF) CX3CL1 levels correlates with cognitive decline in chronic pain patients, we demonstrated that pharmacological blockade of this signaling rescued memory deficits in mice. Mechanistically, integrated transcriptomic and cellular analyses revealed that aberrant CX3CL1-CX3CR1 signaling activated a sequential p38 MAPK-NFκB cascade to upregulate the kinesin motor KIFC2. This KIFC2 upregulation drove a pathogenic remodeling of microglial vesicular trafficking, shifting the secretome toward the massive release of IL-17-enriched small exosomes (<100 nm) that precipitated synaptic deterioration and neurotoxicity. Collectively, our findings describe a comprehensive neuroimmune axis spanning from chemokine receptor activation to Kifc2-dependent exosomal remodeling, unveiling a novel therapeutic strategy for treating pain-associated cognitive comorbidities.

## 2. Results

### 2.1. Elevated CX3CL1 Mediates Chronic Pain-Induced Cognitive Impairment via Microglial CX3CR1

**Figure 1:**
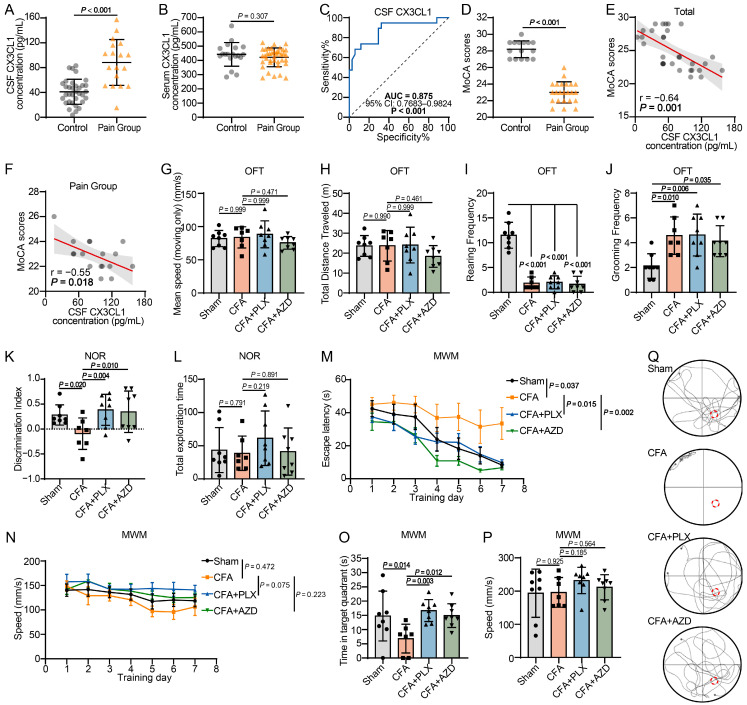
CX3CL1 is elevated in chronic pain and impairs cognition via microglial CX3CR1. (**A**,**B**) ELISA quantification of CX3CL1 concentrations in the CSF ((**A**), Control *n* = 34, Pain *n* = 18) and serum ((**B**), Control *n* = 19, Pain *n* = 39) of clinical cohorts. (**C**) Receiver operating characteristic (ROC) curve evaluating the predictive accuracy of CSF CX3CL1 levels for chronic pain (*n* = 34 for Control, *n* = 18 for Pain). The diagonal dashed line denotes the line of no discrimination. (**D**) MoCA scores reflecting the cognitive status of clinical patients (Control *n* = 15, Pain *n* = 23). (**E**,**F**) Pearson correlation analysis between CSF CX3CL1 concentrations and MoCA scores across the entire clinical cohort (*n* = 30) (**E**) and specifically within the chronic pain group (*n* = 18) (**F**). The solid line represents the linear regression line, with the shaded area indicating the 95% confidence interval. Sample sizes vary across cohorts (**A**,**B**,**D**–**F**) due to the non-interventional use of residual routine clinical specimens; all available unpaired data points were included without arbitrary exclusion. (**G**–**J**) Open field test (OFT) assessing ambulatory speed (**G**), total distance traveled (**H**), rearing frequency (**I**) and grooming frequency (**J**) in Control (*n* = 8), CFA-induced chronic pain (*n* = 7), CFA + PLX5622 (PLX, *n* = 8), and CFA + AZD8797 (AZD, *n* = 8) mouse groups. (**K**,**L**) Novel object recognition (NOR) test evaluating the recognition index (**K**) and total exploration time (**L**) across the four groups. (**M**,**N**) Escape latency to locate the hidden platform (**M**) and corresponding swimming speed (**N**) during the Morris water maze (MWM) training phase. (**O**–**Q**) Probe trial results of the MWM test, showing the time spent in the target quadrant (**O**), overall swimming speed (**P**), and representative swimming trajectories (**Q**). The red dashed circle indicates the former location of the target platform, which was removed during the probe trial. *p*-values were determined by unpaired two-tailed Student’s *t*-test (**A**,**B**,**D**), Pearson correlation analysis (**E**,**F**), one-way ANOVA followed by Tukey’s post hoc test (**G**–**L**,**O**,**P**), or two-way repeated-measures ANOVA with Tukey’s post hoc test (**M**,**N**).

To explore the clinical relevance of CX3CL1 in chronic pain, we quantified its concentrations in the serum and cerebrospinal fluid (CSF) of pain-free controls and patients with osteoarthritis (OA)-associated chronic pain. ELISA analysis indicated that CSF CX3CL1 levels were significantly elevated in chronic pain patients compared to controls (Figure 1A). No significant difference was observed in the serum (Figure 1B). Receiver operating characteristic (ROC) analysis yielded an area under the curve (AUC) of 0.875 (Figure 1C), underscoring the diagnostic efficacy of CSF CX3CL1 as a discriminative biomarker for chronic pain. Further clinical evaluation using Montreal Cognitive Assessment (MoCA) scores revealed a significant decline in cognitive function among patients with chronic pain (Figure 1D). Notably, MoCA scores exhibited a strong negative correlation with CSF CX3CL1 levels globally and within the chronic pain group specifically (Figure 1E,F). Collectively, these clinical findings indicate that elevated CX3CL1 levels in the CSF are closely linked to cognitive impairment in patients with chronic pain.

Given the critical role of microglia in driving cognitive impairment and recognizing the CX3CL1-CX3CR1 axis as a fundamental regulator of their activity, we sought to elucidate its contribution using a complete Freund’s adjuvant (CFA)-induced chronic pain mouse model. Following CFA administration, mice were subjected to either dietary administration of inhibitor PLX5622 to deplete microglia, or intracerebroventricular injection of AZD8797 to block CX3CR1. To rule out the confounding effects of locomotor deficits on cognitive behavioral assessments, we conducted the open field test (OFT). No significant difference in mean ambulatory speed or total distance traveled among the control, CFA-treated, and the two intervention groups (Figure 1G,H), confirming that neither the pain model nor the pharmacological treatments changed their general motor or exploratory capabilities. Concurrently, automated detection of spontaneous nociceptive behaviors suggested successful pain induction [28], characterized by elevated grooming and a substantial decrease in rearing that signaled an aversion to hindlimb weight-bearing (Figure 1I,J). Subsequently, the novel object recognition (NOR) test revealed a significant reduction in the recognition index of CFA-treated mice relative to controls, indicating notable cognitive impairment. Notably, both PLX5622 and AZD8797 treatments largely restored the recognition index (Figure 1K). No variations in exploration time were observed across all four groups (Figure 1L). Consistently, during the 7-day training phase of the Morris water maze (MWM) test, CFA-treated mice exhibited significantly prolonged latencies to find the hidden platform relative to controls. Both microglial depletion by PLX5622 and CX3CR1 blockade by AZD8797 effectively normalized these escape latencies (Figure 1M,N). During the probe trial, CFA mice spent significantly less time in the target quadrant, a deficit that was significantly reversed by either PLX5622 or AZD8797 administration (Figure 1O–Q). Swimming speed remained uniform across all groups throughout the MWM testing. Collectively, these data suggest that CX3CL1-CX3CR1 signaling plays a critical role in chronic pain-associated cognitive impairment, as its pharmacological disruption significantly alleviates these deficits.

### 2.2. The Microglial CX3CL1-CX3CR1 Axis Drives Microglia-Mediated Neurotoxicity

**Figure 2:**
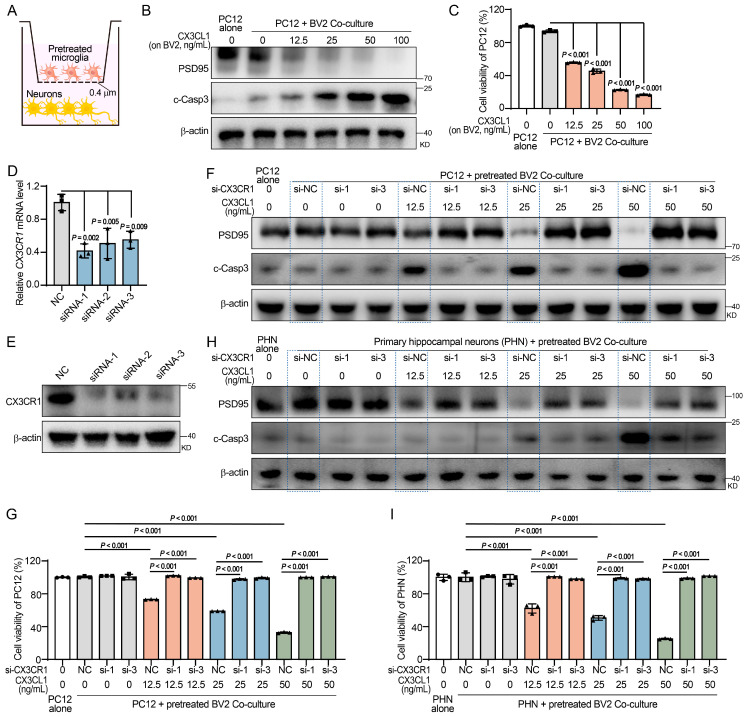
Activation of microglial CX3CL1-CX3CR1 signaling induces neurotoxicity in co-cultured neurons. (**A**) Schematic illustration of the 0.4-μm Transwell system used for co-culturing pre-treated microglia with neuronal cells. (**B**) Western blot analysis of PSD95 and cleaved caspase-3 (c-Casp3) expression in PC12 cells co-cultured with BV2 microglia pre-treated with increasing concentrations of recombinant CX3CL1. (**C**) CCK-8 assay assessing the viability of PC12 cells following co-culture with CX3CL1-stimulated BV2 cells. (**D**,**E**) Validation of CX3CR1 knockdown efficiency in BV2 cells transfected with CX3CR1 siRNA via RT-qPCR (**D**) and Western blotting (**E**). (**F**–**I**) Western blot analysis of PSD95 and cleaved caspase-3 (c-Casp3) expression, alongside CCK-8 cell viability assays, in PC12 cells (**F**,**G**) and primary hippocampal neurons (**H**,**I**). Both neurons were co-cultured with BV2 cells subjected to CX3CR1 siRNA transfection and graded CX3CL1 pre-stimulation. *p*-values were determined by one-way ANOVA followed by Tukey’s post hoc test (**C**,**D**,**G**,**I**).

Given that hippocampal neuronal degeneration and synaptic loss represent the primary pathological substrates of chronic pain-associated cognitive dysfunction, evaluating these paracrine molecular cascades in a physiologically relevant context is imperative [16,26]. Therefore, we established in vitro Transwell co-culture systems utilizing both PC12 cells as a well-characterized neurobiological paradigm and primary murine hippocampal neurons as a bench-marking physiological model (Figure 2A) [29]. Pre-treatment of BV2 cells with increasing concentrations of CX3CL1 elicited pronounced, dose-dependent neurotoxicity in co-cultured PC12 cells. This was evidenced by a progressive loss of the postsynaptic marker PSD95, concomitant with elevated cleaved caspase-3 levels and reduced cellular viability (Figure 2B,C), indicating that CX3CL1-activated microglia exert potent neurotoxic and synaptotoxic effects. Furthermore, we depleted *CX3CR1* expression in BV2 cells using two distinct small interfering RNAs (siRNAs) to validate the requirement for CX3CR1 in this neurotoxicity (Figure 2D,E). Remarkably, targeted knockdown of CX3CR1 abrogated the neurotoxic effects of CX3CL1 pretreatment, rescuing PSD95 expression, reducing cleaved caspase-3, and restoring cell viability in PC12 cells across all CX3CL1 concentrations (Figure 2F,G). Given hippocampal neuronal injury is a crucial contributor to cognitive dysfunction, we co-cultured pre-treated BV2 with primary murine hippocampal neurons. Remarkably, the biological responses of primary hippocampal neurons fully recapitulated the neurotoxic dynamics observed in PC12 cells (Figure 2H,I). Collectively, these in vitro data demonstrate that activation of the microglial CX3CL1-CX3CR1 signaling is a critical driver of microglia-mediated neurotoxicity within the hippocampal microenvironment.

### 2.3. The Microglial CX3CL1-CX3CR1 Signaling Orchestrates an Exosome-Dominant Secretory Pattern via Kifc2 Upregulation

**Figure 3:**
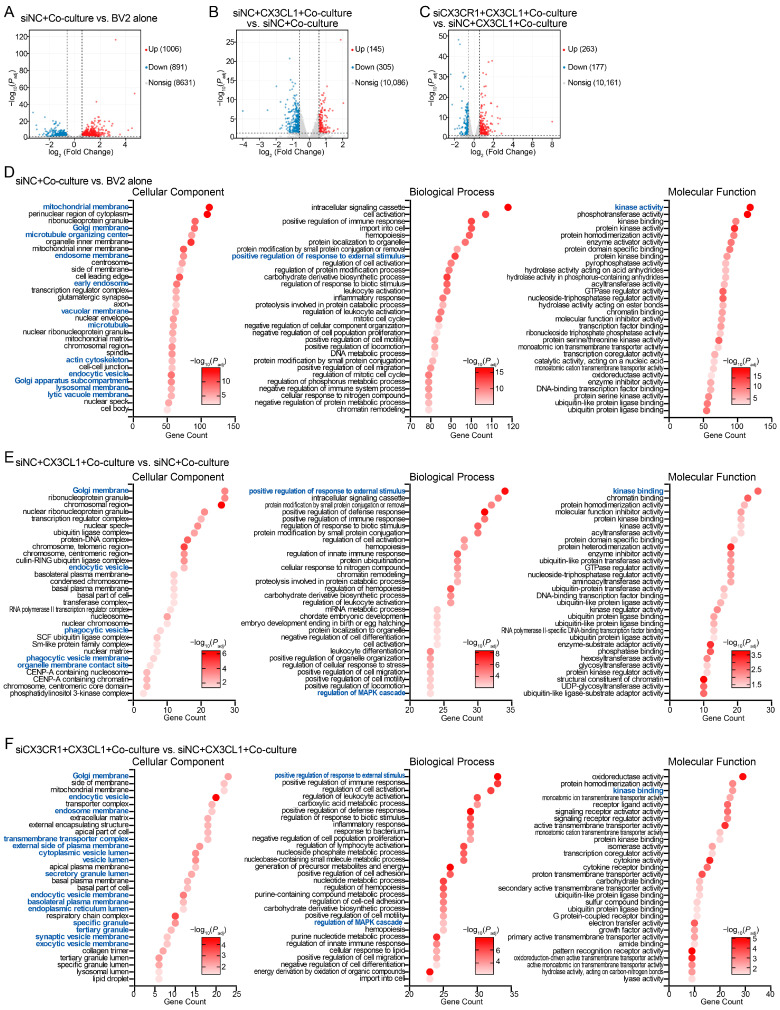
CX3CL1-CX3CR1 signaling drives profound transcriptional reprogramming in microglia. (**A**–**C**) Volcano plots illustrating differentially expressed genes (DEGs) in BV2 cells across three comparisons: co-culture with PC12 cells versus monoculture (**A**), CX3CL1 pre-stimulation prior to co-culture versus vehicle co-culture (**B**), and CX3CR1 siRNA transfection plus CX3CL1 pre-stimulation versus CX3CL1 pre-stimulation alone (**C**). Significant DEGs were defined by ∣log2^(fold change)^∣ > 0.585 and adjusted *p* < 0.05. (**D**–**F**) Bubble plots displaying Gene Ontology (GO) enrichment analysis of the significant DEGs identified in the respective comparisons mentioned above. Vesicle-related and specific signal transduction terms are highlighted in bold blue.

To elucidate mechanisms driving microglia-mediated neurotoxicity, we performed RNA sequencing (RNA-seq) on BV2 cells under various conditions, including monoculture (BV2 alone), co-culture with PC12 cells (siNC + Co-culture), CX3CL1-pretreated prior to co-culture (siNC + CX3CL1 + Co-culture) and CX3CR1-depleted alongside CX3CL1 pretreatment prior to co-culture (siCX3CR1 + CX3CL1 + Co-culture). Through sequential pairwise transcriptomic comparisons, we identified robust sets of differentially expressed genes (DEGs) delineating a progressive phenotypic transition (Figure 3A–C). Compared to monoculture, co-culturing induced substantial transcriptomic alterations in BV2 cells, with DEGs primarily enriched in Gene Ontology (GO) terms associated with endolysosomal trafficking, cytoskeletal remodeling, and initial immune activation (Figure 3D). Upon CX3CL1 stimulation, these co-cultured microglia underwent profound molecular reprogramming. Beyond extensive vesicular transport dynamics, this state was characterized by the involvement of transcriptional and epigenetic regulation alongside an amplified innate immune cascade (Figure 3E). Importantly, targeted knockdown of CX3CR1 perturbed this transcriptomic change, specifically impacting gene sets governing hyperactive secretory phenotype and immunometabolic reprogramming (Figure 3F). Collectively, these transcriptomic alterations suggest that CX3CL1-CX3CR1 signaling triggers notable transcriptional reprogramming in microglia to orchestrate a highly reactive immune state and an aggressive vesicular secretory phenotype.

**Figure 4:**
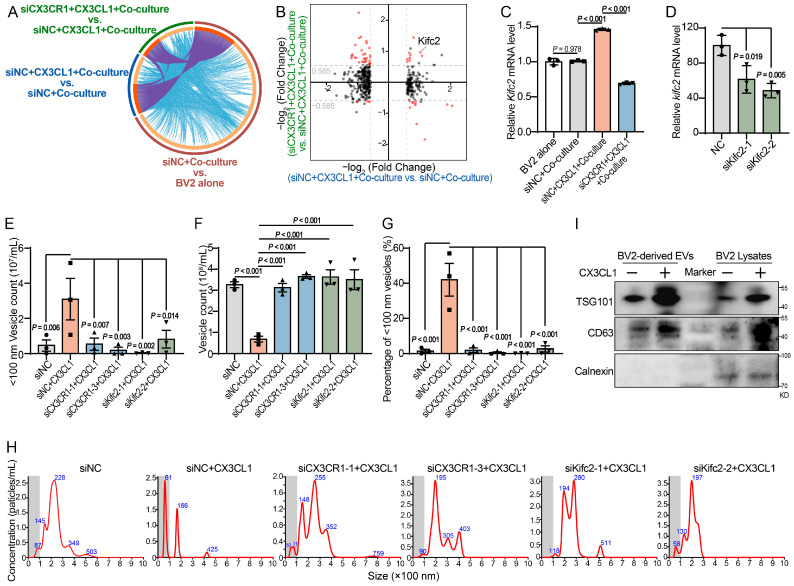
Microglial CX3CL1-CX3CR1 activation promotes small exosome secretion via *Kifc2* upregulation. (**A**) Circos plot demonstrating the functional similarities among the significant DEGs identified in the three transcriptomic comparisons: co-culture with PC12 cells versus monoculture (brick red), CX3CL1 pre-stimulation prior to co-culture versus vehicle co-culture (indigo blue), and CX3CR1 siRNA transfection plus CX3CL1 pre-stimulation versus CX3CL1 pre-stimulation alone (forest green). Purple lines connect directly shared DEGs, whereas blue lines indicate extensive functional interconnectivity among non-overlapping DEGs. (**B**) Quadrant scatter plot comparing the fold changes of DEGs between the CX3CL1 pre-stimulated vs. control group, and the CX3CR1-depleted + CX3CL1 pre-stimulated vs. CX3CL1 pre-stimulated group. (**C**) RT-qPCR quantification of *Kifc2* mRNA levels in BV2 cells under monoculture, co-culture, CX3CL1 pre-stimulated co-culture, and CX3CR1-depleted + CX3CL1 pre-stimulated co-culture conditions. (**D**) RT-qPCR validation of *Kifc2* knockdown efficiency in BV2 cells transfected with *Kifc2* siRNA. (**E**–**H**) Nanoparticle tracking analysis (NTA) of BV2-derived extracellular vesicles following CX3CL1 stimulation or transfection with *Cx3cr1* or *Kifc2* siRNAs, displaying the absolute concentration of small exosomes (<100 nm) (**E**), total EV concentration (**F**), relative proportion of small exosomes (**G**), and overall size distribution profile (**H**). (**I**) Western blot analysis of canonical positive exosome markers (CD63 and TSG101) and the negative control marker (Calnexin) in EV fractions and whole cell lysates and purified EV fractions under control or CX3CL1-stimulated conditions. *p*-values were determined by one-way ANOVA followed by Tukey’s post hoc test (**C**–**G**).

Furthermore, Circos plot analysis using Metascape online database revealed a high degree of overlap among the DEGs across the three comparative groups (Figure 4A). In addition to directly shared DEGs (purple lines), non-overlapping DEGs exhibited extensive functional interconnectivity (blue lines). This functional convergence underscores that the distinct experimental treatments drive overlapping biological pathways, thereby validating the robustness of the downstream mechanisms engaged by the CX3CL1-CX3CR1 signaling. To identify specific downstream effectors, we applied a quadrant scatter plot analysis to compare the fold changes of DEGs between CX3CL1-pretreated and control co-cultured cells against those between CX3CR1-depleted and CX3CL1-pretreated co-cultured cells (Figure 4B). This analysis mapped a massive subset of responsive genes whose CX3CL1-driven upregulation was suppressed by CX3CR1 knockdown. Among these dual-significant DEGs, Kifc2 was prioritized for further investigation, as its prominent marginal position on the plot reflected marked transcriptional responsiveness. Subsequent RT-qPCR in co-cultured BV2 cells validated these RNA-seq findings, indicating that CX3CL1-induced upregulation of *Kifc2* mRNA was effectively abolished upon CX3CR1 depletion (Figure 4C). KIFC2 is a kinesin superfamily molecular motor and previous studies show that KIFC2 mediates the microtubule-dependent retrograde transport of multivesicular body (MVB)-like organelles [30,31]. This canonical role aligns with our GO enrichment analysis, which highlighted prominent alterations in endolysosomal and vesicular transport dynamics. Because MVB transport is required for exosome release, we hypothesized that the CX3CL1-CX3CR1-Kifc2 axis directly regulates microglial vesicle secretion. Nanoparticle tracking analysis (NTA) of BV2-derived EVs showed that CX3CL1 stimulation induced a distinct shift in EV size distribution, characterized by the emergence of a prominent peak at <100 nm (Figure 4E–H). Pathological stress preferentially releases exosomal vesicles distributed under 100 nm [24]. Utilizing this 100 nm threshold to capture this disease-associated phenotype, we found that while the total number of secreted EVs decreased, both the absolute number and relative proportion of exosomes (<100 nm) remarkably increased. Furthermore, targeted knockdown of either CX3CR1 or KIFC2 effectively abolished this shift. Western blot analyses were performed to rigorously validate EV purity and identity. The negative control Calnexin was exclusively detected in whole cell lysates, confirming negligible cellular contamination in the EV isolates. In contrast, the canonical markers CD63 and TSG101 were highly enriched in EV fractions and further elevated by CX3CL1 stimulation (Figure 4I). Taken together, these data demonstrate that CX3CL1-CX3CR1 signaling fundamentally drives a highly reactive, exosome-dominant secretory phenotype in microglia by upregulating *Kifc2*.

### 2.4. The CX3CL1-CX3CR1 Signaling Drives IL-17-Enriched Exosome Secretion and Subsequent Neurotoxicity via the p38 MAPK-NF-κB-Kifc2 Axis

**Figure 5:**
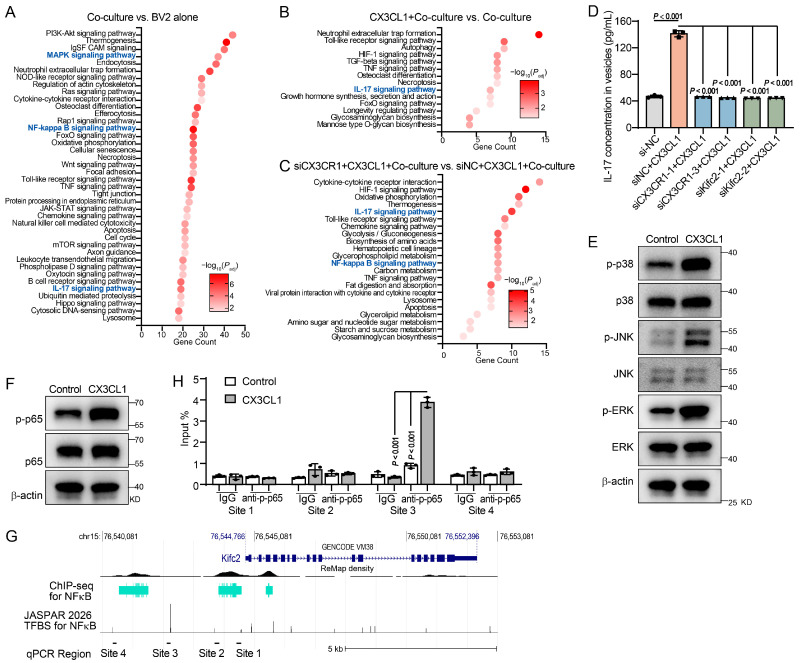
The microglial CX3CL1-CX3CR1 signaling activates MAPK/NF-κB pathways and triggers the secretion of IL-17-enriched exosomes. (**A**–**C**) KEGG pathway enrichment analysis of the significant DEGs identified in three comparisons: co-culture with PC12 cells versus monoculture (**A**), CX3CL1 pre-stimulation prior to co-culture versus vehicle co-culture (**B**), and CX3CR1 siRNA transfection plus CX3CL1 pre-stimulation versus CX3CL1 pre-stimulation alone (**C**). Specific signal transduction terms are highlighted in bold blue. (**D**) ELISA quantification of IL-17 concentrations in EVs secreted by BV2 cells subjected to CX3CL1 stimulation, CX3CR1 siRNA, or *Kifc2* siRNA transfection. (**E**,**F**) Western blot analysis of MAPK (p38, ERK, JNK) and NF-κB (p65) phosphorylation status in BV2 cells following stimulation with 50 ng/mL CX3CL1. (**G**) Predicted and experimental NF-κB binding loci within the *Kifc2* locus, cross-referenced using ReMap ChIP-seq metadata and JASPAR motif analysis. (**H**) ChIP-qPCR assay validating p-p65 binding to the *Kifc2* promoter. *p*-values were determined by one-way ANOVA followed by Tukey’s post hoc test (**D**,**H**).

To characterize the payloads of Kifc2-mediated exosomes and delineate the upstream intracellular cascades, we performed KEGG pathway enrichment analysis on the identified DEGs. Across the three comparative groups, the IL-17, MAPK and NF-κB signaling pathways emerged as consistently enriched (Figure 3E,F and Figure 5A–C). The conspicuous enrichment of the IL-17 signaling suggested its role as a specific constituent of the Kifc2-mediated exosomal cargo. Indeed, ELISA analysis of BV2-derived EVs revealed a marked increase in IL-17 protein content following CX3CL1 stimulation (Figure 5D). Knockdown of either CX3CR1 or KIFC2 significantly abrogated this EV-specific IL-17 accumulation, suggesting that the CX3CL1-CX3CR1-KIFC2 axis actively dictates the packaging and enrichment of IL-17 into exosomes. Moreover, immunoblotting analysis confirmed that CX3CL1 stimulation obviously induced the phosphorylation of MAPK components p38, JNK and ERK, as well as the NF-κB subunit p65 in BV2 cells (Figure 5E,F). Supporting a direct regulatory link, ReMap ChIP-seq metadata derived from murine tissues revealed direct binding of NF-κB to the *Kifc2* promoter region (Figure 5G). Collectively, these findings suggest a regulatory axis in microglia where CX3CL1-CX3CR1 signaling activates the MAPK and NF-κB pathways to transcriptionally upregulate *Kifc2*, thereby driving the preferential secretion of IL-17-loaded exosomes. To validate this direct regulation, we cross-referenced these datasets with JASPAR motif predictions to identify potential p65 binding sites, and subsequently performed a ChIP-qPCR assay. Data showed that the anti-p-p65 antibody yielded a marked enrichment signal at binding site 3 relative to the IgG control under baseline conditions. Remarkably, CX3CL1 stimulation further amplified the recruitment and enrichment of p-p65 at this specific locus (Figure 5H). Collectively, these findings demonstrate a regulatory axis in microglia where CX3CL1-CX3CR1 signaling activates the MAPK and NF-κB pathways to transcriptionally upregulate Kifc2, thereby driving the preferential secretion of IL-17-loaded exosomes.

**Figure 6:**
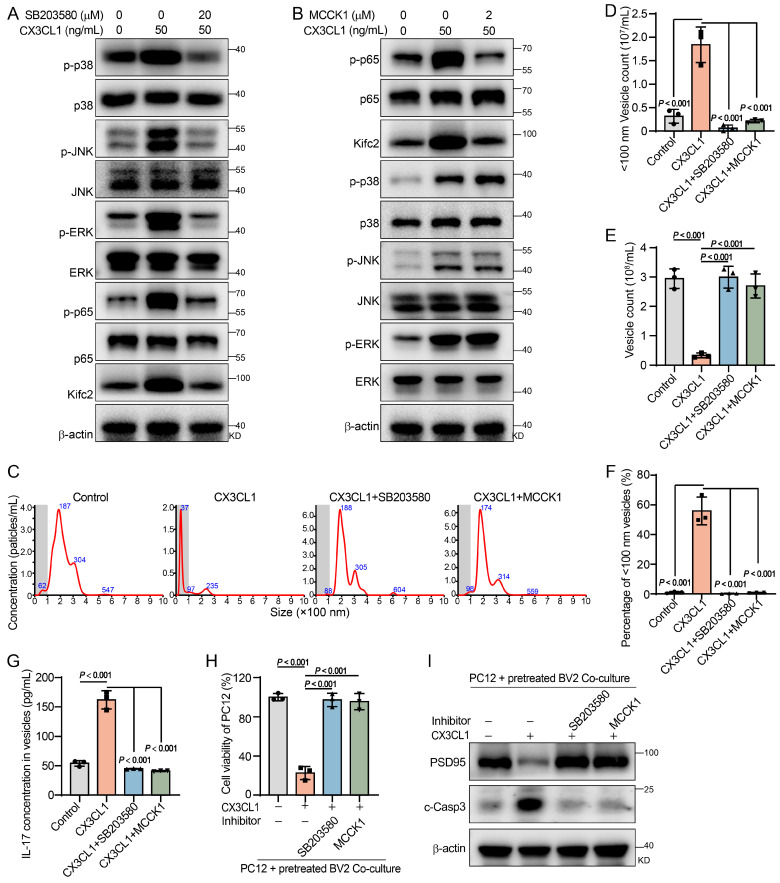
The microglial CX3CL1-CX3CR1 signaling induces neurotoxicity by promoting IL-17-enriched exosome release via the p38 MAPK-NF-κB-KIFC2 axis. (**A**,**B**) Western blot analysis of p38 MAPK-NF-κB phosphorylation and KIFC2 expression in BV2 cells treated with CX3CL1, in the presence of the p38 inhibitor SB203580 (20 μM) or the NF-κB inhibitor MCCK1 (2 μM). (**C**–**F**) NTA characterization of EVs secreted by BV2 cells treated with CX3CL1 in combination with SB203580 or MCCK1, displaying representative size distribution profiles (**C**), absolute concentration of small exosomes (<100 nm) (**D**), total EV concentration (**E**), and the relative proportion of exosomes (**F**). (**G**) ELISA quantification of IL-17 concentrations in EVs secreted by BV2 cells following the indicated inhibitor treatments. (**H**) CCK-8 assay assessing the viability of PC12 cells co-cultured with BV2 cells pre-treated with CX3CL1, SB203580, or MCCK1. (**I**) Western blot analysis of PSD95 and cleaved caspase-3 (c-Casp3) expression in PC12 cells co-cultured with similarly pre-treated BV2 cells. *p*-values were determined by one-way ANOVA followed by Tukey’s post hoc test (**D**–**H**).

Further pharmacological validation of this axis was achieved by pretreating BV2 cells with the p38 MAPK inhibitor SB203580 or the NF-κB inhibitor MCCK1 prior to CX3CL1 stimulation. SB203580 effectively blunted the CX3CL1-evoked activation of the p38 MAPK pathway, along with a concomitant reduction in the phosphorylation of JNK and ERK, which likely reflects potential multi-target profiles of the inhibitor and the dense crosstalk among MAPK subnetworks [32,33]. Crucially, this robust attenuation of the upstream MAPK superfamily subsequently abolished downstream NF-κB activation and suppressed KIFC2 protein upregulation (Figure 6A). Conversely, MCCK1 selectively blocked NF-κB activation and KIFC2 upregulation without affecting MAPK phosphorylation, positioning MAPK upstream of NF-κB (Figure 6B). Functionally, both inhibitors phenocopied the effects of CX3CR1 or Kifc2 knockdown, effectively abolishing the CX3CL1-induced shift towards exosomes (<100 nm) secretion, restoring total EV numbers, and preventing the exosomal enrichment of IL-17 (Figure 6C–G). To evaluate the functional relevance of this signaling axis in microglia-mediated neurotoxicity, we applied these inhibitors in the BV2-PC12 co-culture system. Pharmacological blockade of either p38 or NF-κB in CX3CL1-pretreated BV2 cells effectively protected co-cultured PC12 cells, restoring cell viability and PSD95 expression while reducing cleaved caspase-3 levels (Figure 6H,I). Thus, the microglial CX3CL1-CX3CR1 signaling orchestrates neurotoxicity by activating a p38 MAPK-NF-κB pathway that upregulates KIFC2, ultimately promoting the secretion of IL-17-enriched exosomes.

### 2.5. In Vivo Validation of the Microglial CX3CL1-CX3CR1-p38-NF-κB-KIFC2 Neurotoxic Axis

**Figure 7:**
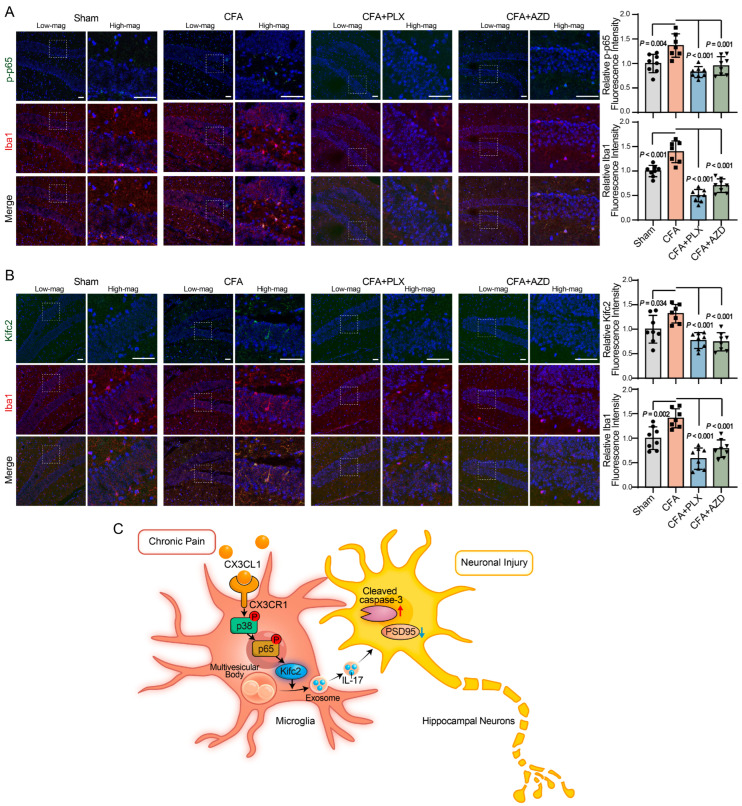
In vivo validation of the microglial CX3CL1-CX3CR1-p38 MAPK-NF-κB-KIFC2 neurotoxic axis in the hippocampus. (**A**,**B**) Representative immunofluorescence images and corresponding quantification showing the expression of Iba1, p-NF-κB (p-p65) (**A**), and Kifc2 (**B**) in the hippocampal dentate gyrus of Control (*n* = 8), CFA (*n* = 7), CFA + PLX5622 (PLX, *n* = 8), and CFA + AZD8797 (AZD, *n* = 8) mice. For the representative images, low-magnification overviews are shown on the left, with white dashed boxes outlining the regions of interest that are displayed at higher magnification on the right. Scale bars = 100 μm. (**C**) A schematic model delineating the proposed mechanism: aberrant activation of the CX3CL1-CX3CR1-p38 MAPK-NF-κB-KIFC2 cascade in microglia drives secretome reprogramming, leading to the massive release of IL-17-enriched exosomes that subsequently precipitate neuronal injury. Red upward arrows indicate the up-regulation of cleaved caspase-3, while blue downward arrows indicate the down-regulation of PSD95. *p*-values were determined by one-way ANOVA followed by Tukey’s post hoc test (**A**,**B**).

To validate this signaling axis in vivo, we performed immunofluorescence staining on hippocampal slices from the aforementioned CFA-induced chronic pain mice subjected to either PLX5622-mediated microglial depletion or AZD8797-mediated CX3CR1 blockade. Colocalization analysis with the microglial marker Iba1 revealed significant elevations of phosphorylated p65 (p-p65) and KIFC2 expression in hippocampal microglia of CFA-treated mice (Figure 7A,B). As expected, PLX5622 administration apparently abolished Iba1 immunoreactivity, whereas AZD8797 administration attenuated microglial activation. Crucially, both pharmacological interventions effectively abrogated the CFA-induced increases in microglial p-p65 and KIFC2. In summary, these in vivo data uncover a novel microglial mechanism wherein elevated CX3CL1-CX3CR1 signaling in chronic pain triggers cognitive impairment by activating microglial p38 MAPK-NF-κB-KIFC2 axis, thereby driving the neurotoxic release of IL-17-enriched exosomes (Figure 7C).

## 3. Discussion

Chronic pain is increasingly recognized not merely as an intractable sensory anomaly but as a pervasive neuromodulatory state that drives enduring cognitive impairment. While maladaptive neuron-microglia crosstalk has been implicated in this comorbidity, the specific intracellular signaling cascades and extracellular mediators linking persistent nociception to synaptic deterioration remain elusive. Here, we identify a previously unrecognized neuroimmune axis that mechanistically couples peripheral nociception to cognitive decline. Integrating clinical CSF analyses with in vivo and in vitro models, we identify hyperactivation of the microglial CX3CL1-CX3CR1 signaling as a key contributor to chronic pain-induced cognitive dysfunction. Mechanistically, we delineate a core intracellular cascade whereby sequential p38 MAPK-NF-κB signaling triggers the transcriptional upregulation of the kinesin motor KIFC2 to orchestrate a profound spatial and structural reprogramming of the microglial secretome. This KIFC2-dependent shift drives the massive release of small exosomes (<100 nm) enriched with the pro-inflammatory cytokine IL-17. Collectively, our findings elucidate the molecular mechanism underlying microglia-mediated neurotoxicity and provide a translatable framework for uncoupling chronic pain from its debilitating cognitive comorbidities.

Our clinical observation that elevated CSF CX3CL1 correlates with cognitive decline in chronic pain patients, coupled with the behavioral rescue following CX3CR1 blockade in animal models, clarifies the context-dependent role of this signaling in neuroimmunology. While canonical CX3CL1-CX3CR1 signaling maintains CNS homeostasis and inhibits microglial activation under physiological states [34], accumulating evidence suggests a dysfunctional role during pathology. For instance, elevated circulating CX3CL1 levels have been strongly correlated with mild cognitive impairment [35], and aberrant CX3CL1-CX3CR1 signaling has been directly implicated in synaptic loss and cognitive deficits associated with hypobaric hypoxia, chronic unpredictable stress, and transient ischemic attack [18,36]. Building upon these reports, our data demonstrate that under sustained nociceptive stress, this normally homeostatic axis undergoes a functional inversion, serving as an upstream driver of hippocampal neuronal injury. This finding aligns with the concept that dysregulated neuron-microglia crosstalk is fundamental to chronic pain morbidities [16]. The efficacy of the CX3CR1 antagonist AZD8797 in rescuing spatial and recognition memory highlights the therapeutic potential of targeting this pathway. Furthermore, the strong negative correlation between CSF CX3CL1 levels and MoCA scores positions CX3CL1 as a valuable clinical biomarker for pain-associated cognitive decline. In summary, targeting this pathway offers a promising therapeutic alternative to conventional analgesics, circumventing their potential to exacerbate cognitive decline via off-target effects on neuronal survival and plasticity.

We identified KIFC2 as a key regulator of the microglial secretome. Generally, microglial EVs comprise endosome-derived exosomes and larger, plasma membrane-budded microvesicles [21]. While EVs mediate crucial neuron-microglia crosstalk in pain [37], their biogenesis in hyperactivated microglia remains elusive. Our NTA analysis under chronic pain stress revealed an interesting phenomenon characterized by a precipitous decline in total EV secretion occurring alongside a massive proportional surge in small exosomes (<100 nm). To decipher this, we considered the canonical biophysical properties of KIFC2, notably its involvement in generating the minus-end-directed mechanical force for early endosomal fission [38] and its selective transport of non-degradative MVBs [30,31]. Translating these properties to our model, we propose that KIFC2 overexpression acts as a molecular sorter and driver of fission. Its increased mechanical force promotes endosomal hyper-fission, rapidly maturing early endosomes into heavily invaginated MVBs packed with intraluminal vesicles. By routing these MVBs through KIFC2’s canonical non-degradative pathway, microglia effectively divert pro-inflammatory cargo away from lysosomal degradation, explaining the intracellular accumulation of IL-17 observed in our study. Notably, this KIFC2-driven remodeling explains the observed secretory shift through intracellular resource competition. The production of larger EV subpopulations relies on outward plasma membrane budding, which competes for limited cellular lipid and cytoskeletal resources. By overexpressing KIFC2, hyperactivated microglia forcefully divert these membrane resources inward to fuel endosome-to-MVB hyper-fission. This reallocation of lipids inhibits outward plasma membrane budding, thereby reducing total EV counts. However, upon fusing with the plasma membrane, these highly processed, degradation-evading MVBs release a concentrated, exosome-dominant secretory burst. Thus, KIFC2 transforms the microglial secretome from homeostatic baseline shedding into a specialized vehicle for delivering neurotoxic payloads.

The enrichment of pathogenic IL-17 within small exosomes (<100 nm) expands the current cytokine signaling model, suggesting that pro-inflammatory mediators are not limited to freely soluble forms. Encapsulating cytokines within EVs provides clear biological advantages. The lipid bilayer protects cytokines from extracellular enzymatic degradation, significantly extending their half-life [39,40], and facilitates targeted, long-range trafficking via a repertoire of surface molecules such as specific lectins, tetraspanins, and membrane receptors [41,42]. Upon reaching target cells, EVs bind to or fuse with the plasma membrane, releasing their active cytokine payload directly at the cell surface [40]. This localized release creates a very high focal concentration of cytokines, rendering receptor engagement more damaging than the diffuse distribution of free extracellular molecules.

This vesicular transport system could amplify the inherently destructive nature of IL-17, a well-documented neurotoxin. Neurons in the hippocampus and dorsal root ganglia express high levels of IL-17 receptors, rendering them highly susceptible to IL-17-mediated injury [43,44,45]. Receptor activation triggers a series of neurodegenerative events, including severe endoplasmic reticulum stress via the Act1-IRE1-caspase-12 axis [46] and increases oxidative stress [45]. Beyond direct cytotoxicity, IL-17 acts as a strong inhibitor of synaptic function. It blocks hippocampal long-term potentiation (LTP), reduces adult neurogenesis, and promotes abnormal complement-mediated synaptic pruning, leading to severe cognitive decline [47]. Concurrently, IL-17 modulates neuronal excitability by increasing excitatory postsynaptic currents and suppressing inhibitory networks, a dual action pivotal in neuropathic and cancer-associated pain [44,48]. The neurodestructive nature of IL-17 is further underscored by clinical and preclinical evidence, where meta-analyses reveal significant serum IL-17 elevations in AD and ALS [49], and its neutralization effectively halts neuronal apoptosis, protects synaptic integrity, and reverses cognitive decline [47,50,51,52]. In our study, hyperactivated microglia used this exosomal transport mechanism to deliver IL-17 through a highly concentrated pathway. This concentrated delivery explains the extensive neuronal injury observed in our co-culture models, characterized by a marked loss of synaptic PSD95 and the activation of caspase-3-dependent apoptosis. Integrating our findings with previous literature suggests that CX3CL1-driven secretion of IL-17-enriched exosomes represents a critical neurotoxic axis driving the cognitive impairment associated with chronic pain.

Interestingly, our behavioral tracking revealed that neither PLX5622-mediated pan-microglial depletion nor intracerebroventricular AZD8797-mediated CX3CR1 blockade normalized the CFA-induced alterations in rearing and grooming frequencies (Figure 1I,J). In the CFA model, persistent nociceptive drive is maintained by a distributed network that includes peripheral immune activation and primary-afferent sensitization, neutrophil/macrophage infiltration within the dorsal root ganglia, central sensitization within the spinal dorsal horn, and supraspinal circuit adaptations [53,54,55,56]. Within this pathophysiological framework, the contribution of microglial signaling is context-dependent and modulatory rather than absolute [57,58]; for instance, intraplantar CFA triggers comparatively weaker spinal microglial reactivity than arthritic models, and suppressing spinal microglia often yields only partial, incomplete alleviation of hypersensitivity [59,60,61,62]. Consequently, depleting microglia or blocking central CX3CR1 signaling is unlikely to abolish the robust peripheral and spinal generators of ongoing inflammatory nociception. Additionally, there is a functional and resolution asymmetry between pain modalities. Evoked assays such as von Frey and Hargreaves employ controlled stimuli with low-variance readouts and are highly sensitive to subtle threshold shifts. Conversely, spontaneous ethological metrics index ongoing pain and its affective–motivational consequences but typically act as high-threshold measures that are relatively insensitive to partial pathway modulation, given that residual nociceptive drive often suffices to maintain weight-bearing aversion and somatic distress [63]. Consistent with this resolution asymmetry, quantitative neuroethology has shown that analgesic regimens capable of restoring evoked thresholds can still fail to normalize spontaneous pain signatures [28]. Thus, the absence of changes in rearing and grooming most likely reflects metric sensitivity rather than a lack of microglial involvement. Importantly, the rescue of memory deficits despite minimal effects on spontaneous pain behaviors supports a potential dissociation between nociceptive drive and the hippocampal mechanisms of cognitive impairment, consistent with the notion that conventional analgesics may fail to improve cognitive performance [9].

Several limitations of this study should be acknowledged. Although we evaluated ethologically relevant spontaneous pain-like behaviors, traditional evoked nociceptive tests such as the von Frey and Hargreaves assays were not included to complement the sensory profiling of this neuroimmune axis. Given the distinct sensitivity profiles of evoked versus spontaneous measures, microglia-targeted interventions may exert effects that are not captured by the rearing and grooming metrics assessed here. Additionally, while differentiated PC12 cells serve as a validated paradigm for signaling research, our downstream pharmacological rescue experiments using SB203580 and MCCK1 were executed exclusively within this cell line framework rather than being paralleled in primary neuronal cultures or ex vivo organotypic slice models. Furthermore, while genetic knockdown experiments provided indirect causal evidence linking exosomal IL-17 accumulation to synaptotoxicity, this functional cascade lacks direct verification via targeted molecular neutralization assays. Future investigations integrating complementary evoked pain models, primary neuronal systems, and in vivo neutralizing antibody deliveries will be essential to fully delineate the therapeutic window and clinical viability of targeting the exosomal CX3CL1-KIFC2-IL-17 axis.

In summary, we identify the CX3CL1-p38 MAPK-NF-κB-KIFC2 axis as a key regulator of microglial exosomal reprogramming. By mapping this neuroimmune axis from receptor signaling to aberrant vesicular trafficking, we described a detailed mechanism of microglia-mediated neurotoxicity. These insights provide a molecular framework for decoupling chronic pain from its severe cognitive comorbidities, offering new avenues for therapeutic intervention.

## 4. Materials and Methods

### 4.1. Animals

Six-week-old C57BL/6 mice (weighing 20–22 g) were purchased from Baijin Biotechnology Co., Ltd. (Hefei, China). Mice were housed in specific pathogen-free facilities under a 12 h light/dark cycle. The room temperature and humidity were strictly controlled. Animals had free access to standard food and water. All animal experiments and reporting strictly adhered to the ARRIVE guidelines 2.0. To mirror the sex distribution of our clinical cohort, both male and female mice were included. For each experiment, the initial sample size assignment per cohort was *n* = 8 (4 females and 4 males) for the Control, CFA + PLX5622, and CFA + AZD8797 groups. Initially containing *n* = 8 mice (4 females, 4 males), the CFA group was reduced to *n* = 7 (4 females, 3 males) for final analysis after one male was humanely euthanized due to postoperative complications. Mice were randomly allocated to the control and treatment groups using a computer-generated randomization sequence. To minimize potential confounding factors, behavioral assessments and subsequent tissue data analysis were performed by investigators who were completely blinded to the group allocations. Following a one-week acclimation period, we established the chronic inflammatory pain model [64]. Briefly, mice were anesthetized with 2% isoflurane. They then received a 50 μL subcutaneous injection of CFA (F5881, Sigma-Aldrich, St. Louis, MO, USA) into the plantar surface of the hind paw. Control mice received the same volume of sterile saline. Mice were monitored in a heated recovery chamber until fully awake. All animal experimental procedures were approved by the Institutional Animal Care and Use Committee of Anhui Medical University (Anhui, China; Approval No. LLSC20231950; 16 May 2023). The studies complied with the National Institutes of Health (NIH) Guide for the Care and Use of Laboratory Animals.

### 4.2. Pharmacological Interventions

To selectively deplete microglia, mice were fed a diet containing the CSF1R inhibitor PLX5622 (HY-1141153C, MedChemExpress, Monmouth Junction, NJ, USA) for one week before pain induction [65]. Daily food consumption was continuously monitored to ensure consistent drug dosing. For continuous intracerebroventricular (i.c.v.) administration, ALZET osmotic pumps (Cupertino, CA, USA) were employed. The pumps were loaded with either AZD8797 (HY13848, MCE, formulated in a vehicle of 30% 2-hydroxypropyl-β-cyclodextrin) or the vehicle alone, and the assembled pump-cannula systems were primed by incubation in sterile saline at 37 °C for 16 h to ensure immediate drug delivery post-implantation. One day before CFA injection, mice were anesthetized with isoflurane and fixed in a stereotaxic frame. Following cranial exposure, an infusion cannula was stereotaxically implanted into the right lateral ventricle (coordinates relative to bregma: AP −0.4 mm, ML +1.0 mm, DV −2.5 mm) and firmly anchored to the skull using dental cement. Concurrently, a subcutaneous pocket was bluntly dissected along the dorsal flank to house the osmotic pump, which was connected to the i.c.v. cannula. Intraoperatively, local infiltration anesthesia was achieved by administering 0.08% bupivacaine (~100 μL per site) into both the cranial incision and the dissected subcutaneous pocket. The incisions were meticulously sutured and disinfected, after which mice were monitored in a heated recovery chamber until fully awake before being returned to their home cages. For post-operative analgesia, acetaminophen (5 mg/mL) was administered via drinking water prepared in a 2% sucrose vehicle from 1 day pre-surgery until 2 days post-surgery, adapted from established protocols [66]. Medicated water was housed in light-shielded water bottles to prevent photodegradation and was fully refreshed every 24 h. Daily water consumption and body weight were carefully monitored to verify palatability, ensure appropriate fluid intake, and prevent potential drug-related toxicity.

### 4.3. Open Field Test (OFT)

Behavioral tests started two weeks after CFA injection. To rule out any confounding effects of the CFA-induced paw inflammation on subsequent cognitive assessments, we performed the open field test (OFT) first to evaluate general locomotion and exploratory behavior and ongoing spontaneous pain-like behaviors. Before the test, mice were habituated to the testing room for 1 h prior to the assay. Each mouse was placed in the center of a white open-field arena (50 × 50 × 50 cm, with a defined 25 × 25 cm central zone) and allowed to explore freely for 5 min. An automated video tracking system (Xinsoft Information Technology Co., Ltd., Shanghai, China) was used to record and automatically quantify travel tracks, speed, and specific non-evoked spontaneous pain-like behaviors. The behavioral paradigms for spontaneous pain were operationally standardized such that rearing frequency was quantified as the total number of vertical exploratory bouts where the animal stood entirely on its hindpaws with both forepaws clear of the ground, where a substantial decrease in rearing serves as a highly sensitive indicator of weight-bearing aversion. Concurrently, grooming frequency was defined and tracked as continuous repetitive bouts of fur licking, body scratching, and face washing, with elevated grooming signifying baseline somatic distress and affective-motivational pain processing [28]. Between trials, to remove olfactory cues the arena was thoroughly cleaned with 75% ethanol.

### 4.4. Novel Object Recognition (NOR) Test

The NOR test started on the same day after the OFT. The experiment lasted for three consecutive days in the open-field arena. Following a 10 min habituation session on Day 1, mice underwent the familiarization phase on Day 2, freely exploring two identical objects placed in opposite corners for 10 min. On Day 3 (testing phase), one of the familiar objects was replaced with a novel object. This novel object differed in shape, texture, and color. The exploratory behavior was recorded for 10 min. Baseline preference tests were conducted to confirm that the mice had no intrinsic bias toward either object.

### 4.5. Morris Water Maze (MWM)

The MWM test started on the day after the NOR test. a circular pool with a diameter of 100 cm and a depth of 50 cm was used. The pool was filled with opaque water kept at 22 °C. On Day 0, mice underwent visible platform training. During this phase, the platform was placed 1 cm above the water surface. From Days 1 to 7, mice underwent hidden platform training. This training included 4 trials per day from random starting locations. Mice failing to find the platform within 60 s were manually guided onto it and kept there for 30 s. On Day 8, the platform was removed for a 60 s probe trial. Due to the preceding stereotaxic i.c.v. surgery, healing cranial incisions were protected with a liquid barrier film (3346, 3M Cavilon, St. Paul, MN, USA) prior to each session. After each trial, the mice were immediately dried and their wounds were disinfected. For the spatial training phase of the MWM, escape latency and swimming speed across the 7-day period were analyzed using a two-way repeated measures ANOVA, with Day as the within-subject factor and Group as the between-subject factor, followed by Tukey’s post hoc test for pairwise multiple comparisons.

### 4.6. Cell Culture

The pheochromocytoma cell line PC12 and the microglial cell line BV2 were obtained from the Cell Bank of the Chinese Academy of Sciences (Shanghai, China). BV2 cells were cultured in DMEM (10564011, Invitrogen, Carlsbad, CA, USA). PC12 cells in RPMI 1640 medium (61870036, Invitrogen). Both culture media were supplemented with 10% fetal bovine serum (26170043, Thermo Fisher Scientific, Waltham, MA, USA), 100 U/mL penicillin, and 0.1 mg/mL streptomycin (P1400, Solarbio, Beijing, China). All cells were maintained at 37 °C in a humidified incubator with 5% CO_2_. Before co-culture, BV2 cells received specific pretreatments to study intracellular signaling pathways. For pharmacological inhibition, BV2 cells were pre-incubated with either the p38 inhibitor SB203580 (HY-10256, MedChemExpress, 20 μM) or the NF-κB inhibitor MCCK1 (HY-D0162R, MedChemExpress, 2 μM) for 12 h, followed by stimulation with recombinant murine CX3CL1 (RP01393, ABclonal, Woburn, MA, USA) for 24 h. For gene silencing experiments, BV2 cells were transfected with small interfering RNAs (siRNAs) targeting *Kifc2* or *Cx3cr1* for 36 h, followed by a 24 h stimulation with recombinant CX3CL1. The specific siRNA sequences (5′ to 3′) were as follows: si-*Kifc2*-1: AGCAUGGUGGAGAUCUACATT; si-*Kifc2*-2: CGUUGCUCAUCUACAUCUUTT; si-*CX3CR1*-1: UUGUUCAUGGAGUUGGGGGTT; si-*CX3CR1*-2: CCUUCUUCUUCAUUGGCUUTT; and si-*CX3CR1*-3: AAGCCAAUGAAGAAGAAGGTT. To evaluate paracrine neurotoxic effects, a co-culture system was established using Transwell inserts (0.4-μm pore size PET membranes, Corning, Corning, NY, USA) in 6-well plates. Crucially, to ensure that the downstream changes in PC12 cells were strictly driven by the microglial secretome rather than residual inhibitors or recombinant cytokines, the pre-treated BV2 cells were thoroughly washed and replenished with fresh culture medium immediately before being introduced into the Transwell co-culture system. PC12 cell viability was assessed using the Cell Counting Kit-8 (C0005, TargetMol, Boston, MA, USA).

### 4.7. Exosome Isolation

Conditioned media harvested from cells cultured in medium containing exosome-depleted FBS were first centrifuged at 2000× *g* for 10 min at 4 °C to remove cells. The supernatant was then centrifuged at 10,000× *g* for 30 min at 4 °C to eliminate apoptotic bodies and large debris. Next, the remaining supernatant was ultracentrifuged at 120,000× *g* for 75 min at 4 °C. The resulting exosome pellet was resuspended in PBS. Finally, the samples were stored at −80 °C for later experiments.

### 4.8. ELISA

Clinical serum was isolated by allowing the blood to clot, followed by centrifugation at 3000 rpm for 15 min at 4 °C. Clinical CSF was centrifuged immediately after collection. Both serum and CSF samples were stored at −80 °C. Human CX3CL1 (ab192145, Abcam, Cambridge, UK) and mouse exosomal IL-17 (E-EL-M0047, Elabscience, Houston, TX, USA) concentrations were quantified using commercial ELISA kits according to the manufacturers’ protocols.

### 4.9. Quantitative RT-PCR (RT-qPCR)

Total RNA was isolated using TRIzol reagent (10606ES60, YEASEN) and quantified using a NanoDrop One spectrophotometer (Thermo Fisher Scientific). Following reverse transcription into cDNA, real-time PCR was performed on a Roche LightCycler system. Relative mRNA expression levels were normalized against the control β-actin (*Actb*) using the comparative 2^−ΔΔCt^ method. The specific primer sequences (5′ to 3′) used in this study were as follows: *Actb*, F: CCTTCCTGGGCATGGAGTC, R: TGATCTTCATTGTGCTGGGTG; *Cx3cr1*, F: GAGTATGACGATTCTGAGG, R: CAGACCGAACGTGAAGAGGAG; and *Kifc2*, F: AAGGGAAATATCCGTGTGCTG, R: GTCTAGGCGGAATCGACGATG.

### 4.10. Chromatin Immunoprecipitation (ChIP)-qPCR

ChIP assays were performed in BV2 cells using a commercial ChIP Assay Kit according to the manufacturer’s instructions. Briefly, cells under baseline or CX3CL1-stimulated conditions were cross-linked with 1% formaldehyde and quenched with glycine. Chromatin was isolated and sonicated to generate DNA fragments ranging from 200 to 500 bp. Immunoprecipitation was conducted overnight at 4 °C using an anti-p-p65 antibody or a non-specific rabbit IgG control. Protein A/G magnetic beads were added to capture the immune complexes. After sequential washing, cross-links were reversed, and the eluted DNA was purified. Potential p-p65 binding sites within the *Kifc2* promoter region were predicted using the JASPAR database, and specific real-time PCR primers targeting these loci were synthesized. Quantitative PCR was performed on the purified DNA, and the target gene enrichment was calculated as a percentage of the total input chromatin (% Input). The specific primer sequences (5′ to 3′) used in this study were as follows: site 1, F: GTTATGACCGAAGGGGAGGTC, R: GGAATCCCGCATCAAGACCC; site 2, F: GGCACGGTCCTAGTTATGGC, R: GTAAGCCGGCTGCTCCAATC; site 3, F: GCAAGTCTACCAGGATGCCAA, R: GGCCTGTAAGGTGATTTCCCTG; site 4, F: GGAGCGGGGTTTCGTAGAC, R: AGCGTCTCTACTCGGTGCT.

### 4.11. Western Blotting

Protein lysates were resolved by SDS-PAGE (10% or 12% gels) and transferred to PVDF membranes. Membranes were probed with primary antibodies against CX3CR1 (1:2000, PC1728S, Abmart, Berkeley Heights, NJ, USA), PSD95 (1:2000, 2507S, CST, Danvers, MA, USA), p-p65 (1:1000, 3033T, CST), p-p38 (1:1000, 9211S, CST), p38 (1:1000, T40075S, Abmart), p65 (1:1000, T55034S, Abmart), JNK (1:1000, T40073S, Abmart), p-JNK (1:1000, PN340810S, Abmart), ERK (1:1000, T40071S, Abmart), p-ERK (1:1000, TA1015S, Abmart), TSG101 (1:1000, ab125011, Abcam), CD63 (1:1000, 25682-1-AP, Proteintech, Chicago, IL, USA), Calnexin (Proteintech, 10427-2-AP, 1:500), and Kifc2 (1:1000, PC17066S, Abmart). A 12% gel was used for Cleaved-caspase-3 (1:2000, ET1608-64, HUABIO, Hangzhou, China) due to its lower molecular weight. β-Actin (1:20,000, 20536-1-AP, Proteintech) served as the loading control. To avoid residual signals or protein loss from stripping and reprobing, proteins were analyzed using equal protein amounts resolved simultaneously on parallel gels under identical conditions. All Western blot experiments were performed with at least three independent biological replicates per condition. To quantify the protein expression, densitometric scanning of the protein bands was performed using ImageJ software (version 1.54).

### 4.12. Immunofluorescence

Brain tissues were cryosectioned, fixed in 4% paraformaldehyde for 30 min, permeabilized with 0.3% Triton X-100 for 10 min, and blocked in 2.5% BSA for 40 min at room temperature. Sections were incubated overnight at 4 °C with primary antibodies against Iba1 (1:500, 018-28523, Wako, Osaka, Japan), p-p65 (1:500, 3033T, CST) and Kifc2 (1:500, PC17066S, Abmart). Sections were rinsed with PBS (3 × 5 min) and incubated with fluorophore-conjugated secondary antibodies for 1 h at room temperature.

### 4.13. RNA Sequencing and Data Analysis

BV2 cells were harvested from monoculture (BV2 alone), co-culture with PC12 cells (siNC + Co-culture), CX3CL1-pretreated prior to co-culture (siNC + CX3CL1 + Co-culture) and CX3CR1-depleted alongside CX3CL1 pretreatment prior to co-culture (siCX3CR1 + CX3CL1 + Co-culture) (*n* = 3 per group). Total RNA was extracted via TRIzol reagent (Cat# 15596018, Invitrogen), and quantified via an Agilent 2100 Bioanalyzer (Agilent Technologies, Santa Clara, CA, USA). cDNA libraries were constructed and sequenced on an Illumina platform. Raw reads were trimmed and aligned to the mouse reference genome (GRCm38) via HISAT2. Gene expression levels were quantified as FPKM. DEGs (|log_2_^(fold change)^| > 0.585 and adjusted *p* < 0.05) were selected globally for downstream functional enrichment analyses. Statistical significance for all the GO and KEGG terms was determined by a Benjamini–Hochberg adjusted *p* < 0.05. In the resulting visualization, the color intensity represents the enrichment significance and the gene count indicates the number of DEGs enriched in each term.

### 4.14. Clinical Sample Collection

Inpatients scheduled for elective, non-infectious, and non-malignant orthopedic surgery under spinal anesthesia were recruited from the affiliated hospitals of Anhui Medical University between August 2023 and April 2026. To isolate osteoarthritis (OA)-associated chronic pain mechanisms, strict exclusion criteria were applied. Patients with preexisting central nervous system disorders, systemic autoimmune diseases, or neuropathic pain were excluded from the study. Preoperative cognitive function was assessed using the Montreal Cognitive Assessment (MoCA). Pain severity was assessed using the 10 cm Visual Analog Scale (VAS; 0 = no pain, 10 = worst imaginable pain), recorded preoperatively. To meet non-interventional ethical standards and minimize patient risk, clinical specimens were collected entirely from residual fluids generated during standard medical procedures. No additional blood draws or dural punctures were performed solely for research purposes. Specifically, serum was isolated from surplus routine preoperative blood samples. CSF (~200 μL) was collected from the residual outflow during the mandatory verification of dural puncture before local spinal anesthesia. Given this strict reliance on clinical waste and natural variations in routine clinical workflows, complete sets of paired CSF, serum, and preoperative MoCA evaluations were not obtainable for all recruited patients. Consequently, downstream analyses were conducted using the maximal available sample size for each specific parameter to prevent selection bias. The clinical characteristics of the study participants are summarized in Table S1. This collection and operation of clinical samples was approved by the Ethics Committee of Anhui Medical University (Approval No. 83230538) and conducted in strict accordance with the Declaration of Helsinki. Written informed consent was obtained from all participants.

### 4.15. Statistical Analysis

Data are presented as mean ± standard deviation (SD) from three independent experiments. Representative pictures capturing consistent biological trends were presented. The detailed densitometric quantification results for all Western blots are provided in Table S2. The normality of data distributions and homogeneity of variances were assessed using the Shapiro–Wilk test and Levene’s test, respectively. For data meeting these assumptions, statistical differences were analyzed using one-way or two-way analysis of variance (ANOVA) followed by Tukey’s post hoc test. Statistical analyses were performed using IBM SPSS Statistics 26.0 software. The specific statistical tests applied to individual datasets are detailed in the corresponding figure legends. *p* < 0.05 was considered statistically significant.

## Data Availability

The RNA-seq datasets generated in this study have been deposited in the Genome Sequence Archive (GSA) under the accession number CRA044196. All relevant data and materials are available from the corresponding author upon reasonable request.

## Supplementary Materials

The following supporting information can be downloaded at: https://www.mdpi.com/article/10.3390/ijms27146304/s1.
