# Supplementary material for: Aberrant CX3CL1-CX3CR1 Signaling Reprograms Microglial Exosome Secretion via KIFC2 to Drive Cognitive Impairment in Chronic Pain
Source: Int J Mol Sci. 2026 Jul 15;27(14):6304. doi: 10.3390/ijms27146304 (PMC13412094; doi:10.3390/ijms27146304)
Supplement: Supplementary file 1 [file ijms-27-06304-s001.zip › ijms-4367530-supplementary.pdf]

Supplementary Information

Aberrant CX3CL1-CX3CR1 signaling reprograms microglial exosome secretion via KIFC2 to drive cognitive impairment in chronic pain

Authors: Chen Hu[1], Xinlu Zhang[1], Wei Zhao[1], Wenjun Ke[1], Haoxiang Ma[1], Wenna Sang[1], Qian Gao[1*]

Affiliations:

1 School of Life Science, Anhui Medical University, Hefei, Anhui, China

*** Correspondence**

Qian Gao, Department of Genetics, School of Life Science, Anhui Medical University, No.81, Meishan Road, Hefei, Anhui Province, China.

E-mail: gaoqian@ahmu.edu.cn

The supplemental information includes the following:

Table S1 Demographic and clinical characteristics of the study participants.

Table S2 Western blot quantification and statistical analysis.

Table S1 Demographic and clinical characteristics of the study participants.

Characteristics	Painless Controls	Chronic Pain Patients	*p*-value
Age (years), *mean ± SD*	52.30 ± 7.571 (N = 37)	55.72 ± 8.220 (N= 39)	0.0638[a] (t = 1.884, df = 74)
Sex (Male/Female), *n (%)*	17 (45.95%) / 20 (54.05%)	17 (43.59%) / 22 (56.41%)	0.8364[b] (χ^2 = 0.04264, df = 1)
Pain Severity (VAS), *median (IQR)*	0.0 (0.0-0.0) (N = 37)	5.0 (4.0-5.0) (N= 39)	< 0.001[c] (U = 0)
MoCA Cognitive Domains			
Total Score, *mean ± SD*	28.20 ± 1.014 (N=15)	23.00 ± 1.279 (N=23)	< 0.001[a] (t = 13.24, df = 36)
Visuospatial / Executive function, *median (IQR)*	4.0 (4.0-5.0)	4.0 (4.0-5.0)	> 0.999[c] (U = 171)
Naming, *median (IQR)*	3.0 (3.0-3.0)	2.0 (2.0-3.0)	0.0202[c] (U = 102)
Attention, *median (IQR)*	6.0 (5.0-6.0)	4.0 (4.0-4.0)	< 0.001[c] (U = 6)
Language, *median (IQR)*	3.0 (3.0-3.0)	2.0 (2.0-2.0)	< 0.001[c] (U = 11.50)
Abstraction, *median (IQR)*	2.0 (1.0-2.0)	1.0 (1.0-1.0)	0.0003[c] (U = 68.50)
Delayed recall, *median (IQR)*	5.0 (5.0-5.0)	4.0 (4.0-5.0)	0.0053[c] (U = 85.50)
Orientation, *median (IQR)*	6.0 (6.0-6.0)	5.0 (5.0-5.0)	< 0.001[c] (U = 6)
CX3CL1 Serum Level (pg/mL), *mean ± SD*	442.2 ± 83.02 (N = 19)	421.5 ± 65.89 (N= 39)	0.3072[a] (t = 1.030, df = 56)
CX3CL1 CSF Level (pg/mL), *mean ± SD*	41.15 ± 20.14 (N = 34)	88.37 ± 36.91 (N= 18)	< 0.001[d] (t = 5.044, df = 22.50)

Note: [a] Analyzed by the independent Student's *t*-test;

[b] Analyzed by the Pearson Chi-square test;

[c] Analyzed by the two-tailed Exact Mann–Whitney U test;

[d] Analyzed by Welch's *t*-test due to unequal variances.

Table S2 Western blot quantification and statistical analysis.

Figure 2B						
	alone-0	co-culture-0	co-culture-12.5	co-culture-25	co-culture-50	co-culture-100
PSD95	2253236.333 ±1016501.457	2108288.333 ± 1080182.95	1677783 ±810388.8452	1493791.333 ± 549914.8672	958134.3333 ± 272074.309	514913.3333 ± 211515.3548
C-Casp3	297322.6667 ± 53459.90574	699743.3333 ± 158229.3516	1174700.333 ± 109398.2301	2303966.333 ±621261.1454	3458810.333 ± 1177667.402	3722947.667 ± 622613.3594
b-actin	3854834.667 ±729285.4956	3533929.333 ±689171.552	3184307.667 ±686679.2139	3527373.333 ±1110575.298	3505849 ±1119385.154	3607736 ±714561.0771
PSD95/b-actin	0.631479253 ±0.37305087	0.639810906 ± 0.377771441	0.576013197 ± 0.360249881	0.487220178 ± 0.306826483	0.305038673 ± 0.155529842	0.144518875 ±0.052481205
C-Casp3/b-actin	0.077356468 ± 0.004532677	0.203058275 ± 0.060191509	0.375367035 ± 0.04753941	0.673184515 ± 0.164673399	0.98727494 ±0.120575771	1.044064609 ± 0.140655195
Relative PSD95/b-actin	1 ±0	0.994510664 ±0.178441429	0.902354594 ±0.044205673	0.767536238 ±0.057111065	0.507593261 ± 0.063722948	0.267958096 ±0.102477097
P value (Relative PSD95/b-actin)	alone-0 vs. co-culture-0 (>0.9999), alone-0 vs. co-culture-12.5 (0.7853), alone-0 vs. co-culture-25 (0.0808), alone-0 vs. co-culture-50 (0.0003), alone-0 vs. co-culture-100(<0.0001), co-culture-0 vs. co-culture-12.5(0.821), co-culture-0 vs. co-culture-25(0.091), co-culture-0 vs. co-culture-50 (0.0004), co-culture-0 vs. co-culture-100 (<0.0001), co-culture-12.5 vs. co-culture-25 (0.5111), co-culture-12.5 vs. co-culture-50 (0.0023), co-culture-12.5 vs. co-culture-100 (<0.0001), co-culture-25 vs. co-culture-50 (0.0442), co-culture-25 vs. co-culture-100 (0.0003), co-culture-50 vs. co-culture-100 (0.0691).					
Relative C-Casp3/b-actin	1 ± 0	2.600694192 ± 0.628296566	4.854116842 ± 0.553956489	8.642387229 ± 1.629775404	12.73051987 ± 0.823541562	13.45638285 ± 1.068121708
P value (Relative C-Casp3/b-actin)	alone-0 vs. co-culture-0 (0.3435), alone-0 vs. co-culture-12.5 (0.0028), alone-0 vs. co-culture-25 (<0.0001), alone-0 vs. co-culture-50 (<0.0001), alone-0 vs. co-culture-100 (<0.0001), co-culture-0 vs. co-culture-12.5 (0.0949), co-culture-0 vs. co-culture-25 (<0.0001), co-culture-0 vs. co-culture-50 (<0.0001), co-culture-0 vs. co-culture-100 (<0.0001), co-culture-12.5 vs. co-culture-25 (0.0033), co-culture-12.5 vs. co-culture-50 (<0.0001), co-culture-12.5 vs. co-culture-100 (<0.0001), co-culture-25 vs. co-culture-50 (0.0017), co-culture-25 vs. co-culture-100 (0.0004), co-culture-50 vs. co-culture-100 (0.923).					

Figure 2E				
	NC	siRNA-1	siRNA-2	siRNA-3
CX3CR1	2650070 ± 955613.5009	647847 ± 284547.677	1274795 ± 616001.9098	530442 ± 255127.5229
beta-actin	4477161.667 ± 908378.5445	4077013.333 ± 813967.3406	4306560.667 ± 563263.2153	4761112.667 ± 1140134.58
CX3CR1/ beta-actin	0.617387101 ± 0.255865747	0.171146559 ± 0.095178286	0.310622348 ± 0.170422043	0.115556991 ± 0.056228667
Relative CX3CR1/ beta-actin	1 ± 0	0.2644886 ± 0.075436669	0.475572437 ± 0.117463129	0.186006291 ± 0.029283216
P value (Relative CX3CR1/ beta-actin)	NC vs. siRNA-1 (<0.0001), NC vs. siRNA-2 (<0.0001), NC vs. siRNA-3 (<0.0001), siRNA-1 vs. siRNA-2 (0.0277), siRNA-1 vs. siRNA-3 (0.5614), siRNA-2 vs. siRNA-3 (0.0048).			
Figure 2F				

	alone	0-si-NC	0-si-1	0-si-3	12.5-si-NC	12.5-si-1	12.5-si-3	25-si-NC	25-si-1	25-si-2	50-si-NC	50-si-1	50-si-1
PSD95	1352926.333 ± 393903.3526	1194998 ± 471557.7137	1267352 ± 504678.056	1491377 ± 557409.2436	1072356 ± 380214.1589	1462109 ± 446380.2403	1538047 ± 526113.8049	570929 ± 161739.6374	1682207 ± 620466.9549	1768931 ± 587693.8062	218338 ± 172250.8783	1713968 ± 739488.3538	1599364 ± 547417.3417
C-Casp3	484111.6667 ± 176311.7073	5744516 ± 246380.3236	5783021 ± 214107.824	589816 ± 136390.4962	1445581 ± 382112.776	559962 ± 187171.0534	585762 ± 255836.0738	1879939 ± 523758.5998	657653 ± 237747.7558	613574 ± 100020.8366	2695416 ± 629715.6084	561570 ± 65590.70112	547858 ± 204804.3243
b-actin	2962535.333 ± 1621170.243	2695336.667 ± 1619685.065	2568262.667 ± 1201789.81	2508254.333 ± 948282.6146	2571565.333 ± 1123163.827	2768456.667 ± 1468615.89	2655737.667 ± 1397758.275	2552560.667 ± 908162.1558	2801221.333 ± 1698083.887	2675451.333 ± 1279710.19	2670412.333 ± 1122266.199	2897931 ± 1278897.72	2806546.333 ± 1419481.104
PSD95/b-actin	0.573361922 ± 0.32653037	0.566562547 ± 0.343141449	0.613025413 ± 0.384450099	0.704461182 ± 0.435968304	0.503008173 ± 0.308392619	0.664062481 ± 0.398533019	0.724179899 ± 0.437226899	0.253556051 ± 0.126528913	0.77979271 ± 0.483052734	0.807991793 ± 0.484285904	0.097536542 ± 0.086548847	0.7016978 ± 0.427433017	0.691368303 ± 0.38438136
C-Casp3/b-actin	0.173320989 ± 0.027174202	0.228013289 ± 0.068162125	0.2313781 ± 0.026485954	0.256818924 ± 0.095049615	0.602001453 ± 0.205248549	0.216580955 ± 0.055010882	0.22679165 ± 0.017851475	0.75825598 ± 0.188336866	0.261058827 ± 0.098533168	0.249996819 ± 0.065277081	1.065006233 ± 0.246458942	0.210553032 ± 0.057127379	0.207659282 ± 0.06138918
Relative PSD95/b-actin	1 ± 0	0.976649758 ± 0.095869163	1.020830376 ± 0.127938522	1.220233575 ± 0.170444784	0.873508904 ± 0.116316037	1.147233194 ± 0.101722029	1.248060141 ± 0.115441177	0.46876587 ± 0.07537633	1.329779005 ± 0.149655003	1.40782947 ± 0.155729308	0.161066421 ± 0.091305782	1.222194614 ± 0.161186518	1.224265765 ± 0.065983734
P value (Relative PSD95/b-actin)	alone vs. 0-si-NC (>0.9999), alone vs. 0-si-1 (>0.9999), alone vs. 0-si-3 (0.552), alone vs. 12.5-si-NC (0.9789), alone vs. 12.5-si-1 (0.938), alone vs. 12.5-si-3 (0.3787), alone vs. 25-si-NC (0.0006), alone vs. 25-si-1 (0.0818), alone vs. 25-si-2 (0.0131), alone vs. 50-si-NC (<0.0001), alone vs. 50-si-1 (0.5391), alone vs. 50-si-1 (0.5256), 0-si-NC vs. 0-si-1 (>0.9999), 0-si-NC vs. 0-si-3 (0.4048), 0-si-NC vs. 12.5-si-NC (0.9961), 0-si-NC vs. 12.5-si-1 (0.8508), 0-si-NC vs. 12.5-si-3 (0.2588), 0-si-NC vs. 25-si-NC (0.001), 0-si-NC vs. 25-si-1 (0.0486), 0-si-NC vs. 25-si-2 (0.0073), 0-si-NC vs. 50-si-NC (<0.0001), 0-si-NC vs. 50-si-1 (0.3932), 0-si-NC vs. 50-si-1 (0.3812), 0-si-1 vs. 0-si-3 (0.688), 0-si-1 vs. 12.5-si-NC (0.9378), 0-si-1 vs. 12.5-si-1 (0.979), 0-si-1 vs. 12.5-si-3 (0.5064), 0-si-1 vs. 25-si-NC (0.0003), 0-si-1 vs. 25-si-1 (0.1269), 0-si-1 vs. 25-si-2 (0.0219), 0-si-1 vs. 50-si-NC (<0.0001), 0-si-1 vs. 50-si-1 (0.6755), 0-si-1 vs. 50-si-1 (0.6622),0-si-3 vs. 12.5-si-NC (0.0562), 0-si-3 vs. 12.5-si-1 (0.9999), 0-si-3 vs. 12.5-si-3 (>0.9999), 0-si-3 vs. 25-si-NC (<0.0001), 0-si-3 vs. 25-si-1 (0.9934), 0-si-3 vs. 25-si-2 (0.7604), 0-si-3 vs. 50-si-NC (<0.0001), 0-si-3 vs. 50-si-1 (>0.9999), 0-si-3 vs. 50-si-1 (>0.9999), 12.5-si-NC vs. 12.5-si-1 (0.2485), 12.5-si-NC vs. 12.5-si-3 (0.0295), 12.5-si-NC vs. 25-si-NC (0.0142), 12.5-si-NC vs. 25-si-1												

	(0.0039), 12.5-si-NC vs. 25-si-2 (0.0005), 12.5-si-NC vs. 50-si-NC (<0.0001), 12.5-si-NC vs. 50-si-1 (0.0538), 12.5-si-NC vs. 50-si-1 (0.0513), 12.5-si-1 vs. 12.5-si-3 (0.9968), 12.5-si-1 vs. 25-si-NC (<0.0001), 12.5-si-1 vs. 25-si-1 (0.7892), 12.5-si-1 vs. 25-si-2 (0.3108), 12.5-si-1 vs. 50-si-NC (<0.0001), 12.5-si-1 vs. 50-si-1 (0.9998), 12.5-si-1 vs. 50-si-1 (0.9998), 12.5-si-3 vs. 25-si-NC (<0.0001), 12.5-si-3 vs. 25-si-1 (0.9996), 12.5-si-3 vs. 25-si-2 (0.8969), 12.5-si-3 vs. 50-si-NC (<0.0001), 12.5-si-3 vs. 50-si-1 (>0.9999), 12.5-si-3 vs. 50-si-1 (>0.9999), 25-si-NC vs. 25-si-1 (<0.0001), 25-si-NC vs. 25-si-2 (<0.0001), 25-si-NC vs. 50-si-NC (0.1301), 25-si-NC vs. 50-si-1 (<0.0001), 25-si-NC vs. 50-si-1 (<0.0001), 25-si-1 vs. 25-si-2 (0.9997), 25-si-1 vs. 50-si-NC (<0.0001), 25-si-1 vs. 50-si-1 (0.9944), 25-si-1 vs. 50-si-1 (0.9952), 25-si-2 vs. 50-si-NC (<0.0001), 25-si-2 vs. 50-si-1 (0.7717), 25-si-2 vs. 50-si-1 (0.7835), 50-si-NC vs. 50-si-1 (<0.0001), 50-si-NC vs. 50-si-1 (<0.0001), 50-si-1 vs. 50-si-1 (>0.9999).												
Relative C-Casp 3/b-actin	1 ± 0	1.310985442 ± 0.290670533	1.344692149 ± 0.122035112	1.444827506 ± 0.353679504	3.461521504 ± 0.932037683	1.247244869 ± 0.218906463	1.320485396 ± 0.114957275	4.378791702 ± 0.809802789	1.487885103 ± 0.425154472	1.42575608 ± 0.178392156	6.141676329 ± 0.956353768	1.198854073 ± 0.155439628	1.199260317 ± 0.278698199
P value (Relative PSD 95/b-actin)	alone vs. 0-si-NC (0.9998), alone vs. 0-si-1 (0.9994), alone vs. 0-si-3 (0.9936), alone vs. 12.5-si-NC (<0.0001), alone vs. 12.5-si-1 (>0.9999), alone vs. 12.5-si-3 (0.9997), alone vs. 25-si-NC (<0.0001), alone vs. 25-si-1 (0.9863), alone vs. 25-si-2 (0.9956), alone vs. 50-si-NC (<0.0001), alone vs. 50-si-1 (>0.9999), alone vs. 50-si-1 (>0.9999), 0-si-NC vs. 0-si-1 (>0.9999), 0-si-NC vs. 0-si-3 (>0.9999), 0-si-NC vs. 12.5-si-NC (0.0006), 0-si-NC vs. 12.5-si-1 (>0.9999), 0-si-NC vs. 12.5-si-3 (>0.9999), 0-si-NC vs. 25-si-NC (<0.0001), 0-si-NC vs. 25-si-1 (>0.9999), 0-si-NC vs. 25-si-2 (>0.9999), 0-si-NC vs. 50-si-NC (<0.0001), 0-si-NC vs. 50-si-1 (>0.9999), 0-si-NC vs. 50-si-1 (>0.9999), 0-si-1 vs. 0-si-3 (>0.9999), 0-si-1 vs. 12.5-si-NC (0.0008), 0-si-1 vs. 12.5-si-1 (>0.9999), 0-si-1 vs. 12.5-si-3 (>0.9999), 0-si-1 vs. 25-si-NC (<0.0001), 0-si-1 vs. 25-si-1 (>0.9999), 0-si-1 vs. 25-si-2 (>0.9999), 0-si-1 vs. 50-si-NC (<0.0001), 0-si-1 vs. 50-si-1 (>0.9999), 0-si-1 vs. 50-si-1 (>0.9999), 0-si-3 vs. 12.5-si-NC (0.0014), 0-si-3 vs. 12.5-si-1 (>0.9999), 0-si-3 vs. 12.5-si-3 (>0.9999), 0-si-3 vs. 25-si-NC (<0.0001), 0-si-3 vs. 25-si-1 (>0.9999), 0-si-3 vs. 25-si-2 (>0.9999), 0-si-3 vs. 50-si-NC (<0.0001), 0-si-3 vs. 50-si-1 (>0.9999), 0-si-3 vs. 50-si-1 (>0.9999), 12.5-si-NC vs. 12.5-si-1 (0.0004), 12.5-si-NC vs. 12.5-si-3 (0.0007), 12.5-si-NC vs. 25-si-NC (0.521), 12.5-si-NC vs. 25-si-1 (0.0019), 12.5-si-NC vs. 25-si-2 (0.0013), 12.5-si-NC vs. 50-si-NC (<0.0001), 12.5-si-NC vs. 50-si-1 (0.0003), 12.5-si-NC vs. 50-si-1 (0.0003), 12.5-si-1 vs. 12.5-si-3 (>0.9999), 12.5-si-1 vs. 25-si-NC (<0.0001), 12.5-si-1 vs. 25-si-1 (>0.9999), 12.5-si-1 vs. 25-si-2 (>0.9999), 12.5-si-1 vs. 50-si-NC (<0.0001), 12.5-si-1 vs. 50-si-1 (>0.9999), 12.5-si-1 vs. 50-si-1 (>0.9999), 12.5-si-3 vs. 25-si-NC (<0.0001), 12.5-si-3 vs. 25-si-1 (>0.9999), 12.5-si-3 vs. 25-si-2 (>0.9999), 12.5-si-3 vs. 50-si-NC (<0.0001), 12.5-si-3 vs. 50-si-1 (>0.9999), 12.5-si-3 vs. 50-si-1 (>0.9999), 25-si-NC vs. 25-si-1 (<0.0001), 25-si-NC vs. 25-si-2 (<0.0001), 25-si-NC vs. 50-si-NC (0.0071), 25-si-NC vs. 50-si-1 (<0.0001), 25-si-NC vs. 50-si-1 (<0.0001), 25-si-1 vs. 25-si-2 (>0.9999), 25-si-1 vs. 50-si-NC (<0.0001), 25-si-1 vs. 50-si-1 (0.9999), 25-si-1 vs. 50-si-1 (0.9999), 25-si-2 vs. 50-si-NC (<0.0001), 25-si-2 vs. 50-si-1 (>0.9999), 25-si-2 vs. 50-si-1 (>0.9999), 50-si-NC vs. 50-si-1 (<0.0001), 50-si-NC vs. 50-si-1 (<0.0001), 50-si-1 vs. 50-si-1 (>0.9999),												
Figure 2H													
	alone	0-si-NC	0-si-1	0-si-3	12.5-si-NC	12.5-si-1	12.5-si-3	25-si-NC	25-si-1	25-si-2	50-si-NC	50-si-1	50-si-1
PSD 95	1174117.333 ± 440193.6181	1150623.667 ± 377870.235	1169699 ± 527982.4424	1406445.667 ± 604012.6729	767202.333 ± 290244.613	1181442.667 ± 441461.6512	1208219 ± 449038.9597	360670.333 ± 301792.6337	941724 ± 279210.8774	1327001 ± 711060.2832	177708.667 ± 190528.9259	850914.667 ± 215243.5372	1214461.667 ± 550889.3256
C-Casp 3	573491.333 ± 1930	435986.667 ± 1779	438466.667 ± 2509	365420 ± 293520.48	418688 ± 273268.76	556747.333 ± 4365	521293.667 ± 3931	1034782.333 ± 4602	496324.667 ± 1639	630406 ± 222324.72	2145665.333 ± 7681	611245 ± 129871.57	783884 ± 246376.8

	74.9751	39.6146	66.9161	12	96	59.0099	10.0828	40.6376	95.7755	26	19.6692	16	681
b-actin	2195191 ± 894643.1185	2127185.667 ± 827948.0447	2002859 ± 798960.6558	2084530.667 ± 687018.5855	2253749 ± 818220.0973	2367064 ± 780927.2827	2108708.667 ± 968891.3204	2033869.333 ± 780304.0349	1969391.333 ± 882105.388	2133771.667 ± 1063818.4	1993122.667 ± 992789.2363	1655809 ± 529674.9769	2253928.333 ± 1051485.398
PSD95/b-actin	0.580717425 ± 0.255476629	0.592274841 ± 0.280458636	0.647745162 0.390378752	0.691944523 ± 0.248757165	0.366936561 ± 0.16175767	0.510887162 ± 0.144545748	0.608006584 ± 0.161473957	0.159910772 ± 0.076068338	0.532878286 ± 0.217976272	0.635150361 ± 0.126163211	0.074362215 ± 0.051549743	0.528951519 ± 0.090315615	0.574256465 ± 0.193581469
C-Casp3/b-actin	0.280449501 ± 0.094549072	0.220499573 ± 0.090686931	0.20920382 ± 0.090857576	0.171921974 ± 0.139624702	0.18361591 ± 0.096287782	0.22349088 ± 0.177630252	0.240212682 ± 0.171203975	0.531032975 ± 0.228783024	0.2683545 ± 0.064960273	0.345240006 ± 0.160572474	1.167975047 ± 0.33678462	0.415973956 ± 0.22305834	0.377746243 ± 0.110081411
Relative PSD95/b-actin	1 ± 0	1.013393024 ± 0.050304369	1.05817921 ± 0.198447648	1.217984682 ± 0.149466994	0.629453587 ± 0.042496512	0.921540567 ± 0.147007059	1.109196025 ± 0.231442892	0.30768575 ± 0.160323431	0.932966872 ± 0.093639161	1.177103579 ± 0.285022279	0.144192781 ± 0.099414474	0.988240353 ± 0.266658886	1.019123761 ± 0.104267315
P value (Relative PSD95/b-actin)	alone vs. 0-si-NC (>0.9999), alone vs. 0-si-1 (>0.9999), alone vs. 0-si-3 (0.9042), alone vs. 12.5-si-NC (0.2733), alone vs. 12.5-si-1 (>0.9999), alone vs. 12.5-si-3 (0.9997), alone vs. 25-si-NC (0.0012), alone vs. 25-si-1 (>0.9999), alone vs. 25-si-2 (0.9766), alone vs. 50-si-NC (<0.0001), alone vs. 50-si-1 (>0.9999), alone vs. 50-si-1 (>0.9999), 0-si-NC vs. 0-si-1 (>0.9999), 0-si-NC vs. 0-si-3 (0.9356), 0-si-NC vs. 12.5-si-NC (0.2304), 0-si-NC vs. 12.5-si-1 (>0.9999), 0-si-NC vs. 12.5-si-3 (>0.9999), 0-si-NC vs. 25-si-NC (0.0009), 0-si-NC vs. 25-si-1 (>0.9999), 0-si-NC vs. 25-si-2 (0.9874), 0-si-NC vs. 50-si-NC (<0.0001), 0-si-NC vs. 50-si-1 (>0.9999), 0-si-NC vs. 50-si-1 (>0.9999), 0-si-1 vs. 0-si-3 (0.9896), 0-si-1 vs. 12.5-si-NC (0.1232), 0-si-1 vs. 12.5-si-1 (0.9973), 0-si-1 vs. 12.5-si-3 (>0.9999), 0-si-1 vs. 25-si-NC (0.0004), 0-si-1 vs. 25-si-1 (0.9988), 0-si-1 vs. 25-si-2 (0.9993), 0-si-1 vs. 50-si-NC (<0.0001), 0-si-1 vs. 50-si-1 (>0.9999), 0-si-1 vs. 50-si-1 (>0.9999), 0-si-3 vs. 12.5-si-NC (0.0083), 0-si-3 vs. 12.5-si-1 (0.589), 0-si-3 vs. 12.5-si-3 (0.9997), 0-si-3 vs. 25-si-NC (<0.0001), 0-si-3 vs. 25-si-1 (0.6433), 0-si-3 vs. 25-si-2 (>0.9999), 0-si-3 vs. 50-si-NC (<0.0001), 0-si-3 vs. 50-si-1 (0.8701), 0-si-3 vs. 50-si-1 (0.9466), 12.5-si-NC vs. 12.5-si-1 (0.6098), 12.5-si-NC vs. 12.5-si-3 (0.0554), 12.5-si-NC vs. 25-si-NC (0.4699), 12.5-si-NC vs. 25-si-1 (0.5553), 12.5-si-NC vs. 25-si-2 (0.0174), 12.5-si-NC vs. 50-si-NC (0.0506), 12.5-si-NC vs. 50-si-1 (0.3151), 12.5-si-NC vs. 50-si-1 (0.2137), 12.5-si-1 vs. 12.5-si-3 (0.9643), 12.5-si-1 vs. 25-si-NC (0.0052), 12.5-si-1 vs. 25-si-1 (>0.9999), 12.5-si-1 vs. 25-si-2 (0.7753), 12.5-si-1 vs. 50-si-NC (0.0002), 12.5-si-1 vs. 50-si-1 (>0.9999), 12.5-si-1 vs. 50-si-1 (0.9999), 12.5-si-3 vs. 25-si-NC (0.0002), 12.5-si-3 vs. 25-si-1 (0.9775), 12.5-si-3 vs. 25-si-2 (>0.9999), 12.5-si-3 vs. 50-si-NC (<0.0001), 12.5-si-3 vs. 50-si-1 (0.9991), 12.5-si-3 vs. 50-si-1 (>0.9999), 25-si-NC vs. 25-si-1 (0.0042), 25-si-NC vs. 25-si-2 (<0.0001), 25-si-NC vs. 50-si-NC (0.9875), 25-si-NC vs. 50-si-1 (0.0015), 25-si-NC vs. 50-si-1 (0.0008), 25-si-1 vs. 25-si-2 (0.8203), 25-si-1 vs. 50-si-NC (0.0002), 25-si-1 vs. 50-si-1 (>0.9999), 25-si-1 vs. 50-si-1 (>0.9999), 25-si-2 vs. 50-si-NC (<0.0001), 25-si-2 vs. 50-si-1 (0.9626), 25-si-2 vs. 50-si-1 (0.9905), 50-si-NC vs. 50-si-1 (<0.0001), 50-si-NC vs. 50-si-1 (<0.0001), 50-si-1 vs. 50-si-1 (>0.9999),												
Relative C-	1 ± 0	0.770789801 ±	0.806092104 ±	0.609715634 ±	0.653118819 ±	0.825390325 ±	0.860347965 ±	1.894599698 ±	0.985849505 ±	1.190236335 ±	4.22564347 ±	1.471612328 ±	1.36713709 ±

Casp 3/b-actin		0.078514054	0.39163503	0.366433323	0.206019978	0.529957668	0.447196643	0.399557216	0.136697044	0.258251069	0.285235712	0.534094039	0.11642766
P value (Relative C-Casp 3/b-actin)	alone vs. 0-si-NC (0.9996), alone vs. 0-si-1 (>0.9999), alone vs. 0-si-3 (0.9581), alone vs. 12.5-si-NC (0.9825), alone vs. 12.5-si-1 (>0.9999), alone vs. 12.5-si-3 (>0.9999), alone vs. 25-si-NC (0.1041), alone vs. 25-si-1 (>0.9999), alone vs. 25-si-2 (>0.9999), alone vs. 50-si-NC (<0.0001), alone vs. 50-si-1 (0.8631), alone vs. 50-si-1 (0.973), 0-si-NC vs. 0-si-1 (>0.9999), 0-si-NC vs. 0-si-3 (>0.9999), 0-si-NC vs. 12.5-si-NC (>0.9999), 0-si-NC vs. 12.5-si-1 (>0.9999), 0-si-NC vs. 12.5-si-3 (>0.9999), 0-si-NC vs. 25-si-NC (0.0159), 0-si-NC vs. 25-si-1 (0.9998), 0-si-NC vs. 25-si-2 (0.9321), 0-si-NC vs. 50-si-NC (<0.0001), 0-si-NC vs. 50-si-1 (0.3705), 0-si-NC vs. 50-si-1 (0.6041), 0-si-1 vs. 0-si-3 (>0.9999), 0-si-1 vs. 12.5-si-NC (>0.9999), 0-si-1 vs. 12.5-si-1 (>0.9999), 0-si-1 vs. 12.5-si-3 (>0.9999), 0-si-1 vs. 25-si-NC (0.0216), 0-si-1 vs. 25-si-1 (>0.9999), 0-si-1 vs. 25-si-2 (0.9625), 0-si-1 vs. 50-si-NC (<0.0001), 0-si-1 vs. 50-si-1 (0.445), 0-si-1 vs. 50-si-1 (0.6859), 0-si-3 vs. 12.5-si-NC (>0.9999), 0-si-3 vs. 12.5-si-1 (0.9998), 0-si-3 vs. 12.5-si-3 (0.999), 0-si-3 vs. 25-si-NC (0.0038), 0-si-3 vs. 25-si-1 (0.9678), 0-si-3 vs. 25-si-2 (0.6411), 0-si-3 vs. 50-si-NC (<0.0001), 0-si-3 vs. 50-si-1 (0.1324), 0-si-3 vs. 50-si-1 (0.2668), 12.5-si-NC vs. 12.5-si-1 (>0.9999), 12.5-si-NC vs. 12.5-si-3 (0.9998), 12.5-si-NC vs. 25-si-NC (0.0056), 12.5-si-NC vs. 25-si-1 (0.9874), 12.5-si-NC vs. 25-si-2 (0.7388), 12.5-si-NC vs. 50-si-NC (<0.0001), 12.5-si-NC vs. 50-si-1 (0.1795), 12.5-si-NC vs. 50-si-1 (0.3444), 12.5-si-1 vs. 12.5-si-3 (>0.9999), 12.5-si-1 vs. 25-si-NC (0.0255), 12.5-si-1 vs. 25-si-1 (>0.9999), 12.5-si-1 vs. 25-si-2 (0.9743), 12.5-si-1 vs. 50-si-NC (<0.0001), 12.5-si-1 vs. 50-si-1 (0.4882), 12.5-si-1 vs. 50-si-1 (0.7288), 12.5-si-3 vs. 25-si-NC (0.0342), 12.5-si-3 vs. 25-si-1 (>0.9999), 12.5-si-3 vs. 25-si-2 (0.9882), 12.5-si-3 vs. 50-si-NC (<0.0001), 12.5-si-3 vs. 50-si-1 (0.5692), 12.5-si-3 vs. 50-si-1 (0.8008), 25-si-NC vs. 25-si-1 (0.0935), 25-si-NC vs. 25-si-2 (0.3634), 25-si-NC vs. 50-si-NC (<0.0001), 25-si-NC vs. 50-si-1 (0.9283), 25-si-NC vs. 50-si-1 (0.7592), 25-si-1 vs. 25-si-2 (0.9999), 25-si-1 vs. 50-si-NC (<0.0001), 25-si-1 vs. 50-si-1 (0.8394), 25-si-1 vs. 50-si-1 (0.9645), 25-si-2 vs. 50-si-NC (<0.0001), 25-si-2 vs. 50-si-1 (0.997), 25-si-2 vs. 50-si-1 (>0.9999), 50-si-NC vs. 50-si-1 (<0.0001), 50-si-NC vs. 50-si-1 (<0.0001), 50-si-1 vs. 50-si-1 (>0.9999),												

Figure 6A

	Control	CX3CL1	CX3CL1+SB203580
p-p38	2818908 ± 2805273.211	3881672.333 ± 4014468.769	2016194 ± 2146173.032
p38	7757731 ± 848583.2058	7543544.667 ± 1117109.137	7231015.667 ± 1092251.407
p-JNK	2651426 ± 1100096.889	5219039.333 ± 2934173.793	2606781.333 ± 553009.8587
JNK	4834180.667 ± 697958.8126	4906375.333 ± 880832.5216	5222928 ± 1058595.053
p-ERK	3730302.333 ± 728039.9168	6969209.667 ± 2178407.649	3221640.333 ± 948537.3551
ERK	6216147.333 ± 2181693.834	6006664.667 ± 2438393.217	5510022.667 ± 2208361.828
p-p65	3717052.667 ± 1205922.715	7399482.667 ± 2101481.46	3921779 ± 1399005.631
p65	6508118.667 ± 1221655.908	6614761.667 ± 1427451.171	6157387.667 ± 1137034.008
kifc2	3597242.333 ± 476403.7191	5700597.333 ± 477560.6531	3884298.667 ± 960027.7075
b-actin	5314585.667 ± 1281528.398	5438039 ± 1461276.809	5333953 ± 1265212.377
p-p38/p38	0.35747375 ± 0.354687401	0.499374181 ± 0.511921278	0.27092178 ± 0.282100382
p-JNK/JNK	0.567124227 ± 0.262424119	1.094263869 ± 0.597377074	0.513116235 ± 0.137317146
p-ERK/ERK	0.629235864 ± 0.131555857	1.203938631 ± 0.165072269	0.613975165 ± 0.125998236

p-p65/65	0.60992894 ± 0.311584532	1.196574825 ± 0.557294868	0.677975351 ± 0.338096076
kifc2/b-actin	0.712205791 ± 0.222514502	1.084605123 ± 0.210345101	0.771516647 ± 0.296818726
Relative p-p38/p38	1 ± 0	1.354253298 ± 0.087906069	0.743672092 ± 0.092341584
P value (Relative p-p38/p38)	Control vs. CX3CL1 (0.0026), Control vs. CX3CL1+SB203580 (0.0125), CX3CL1 vs. CX3CL1+SB203580 (0.0001).		
Relative p-JNK/JNK	1 ± 0	1.865665884 ± 0.208377234	0.985744276 ± 0.265818457
P value (Relative p-JNK/JNK)	Control vs. CX3CL1 (0.0039), Control vs. CX3CL1+SB203580 (0.9956), CX3CL1 vs. CX3CL1+SB203580 (0.0036).		
Relative p-ERK/ERK	1 ± 0	1.930999575 ± 0.127610567	0.981790293 ± 0.150609848
P value (Relative p-ERK/ERK)	Control vs. CX3CL1 (0.0001), Control vs. CX3CL1+SB203580 (0.9792), CX3CL1 vs. CX3CL1+SB203580 (0.0001)		
Relative p-p65/65	1 ± 0	1.984552123 ± 0.145795087	1.107180081 ± 0.159196387
P value (Relative p-p65/65)	Control vs. CX3CL1 (0.0002), Control vs. CX3CL1+SB203580 (0.5739), CX3CL1 vs. CX3CL1+SB203580 (0.0003)		
Relative kifc2/b-actin	1 ± 0	1.572523157 ± 0.242266398	1.063285622 ± 0.09887917
P value (Relative kifc2/b-actin)	Control vs. CX3CL1 (0.0084), Control vs. CX3CL1+SB203580 (0.8679), CX3CL1 vs. CX3CL1+SB203580 (0.0145).		
Figure 6B			
	Control	CX3CL1	CX3CL1+SB203580
p-p38	2293288 ± 905008.8087	4774324.777 ± 1394010.869	4945893.889 ± 826034.2401
p38	5553416.667 ± 1016832.575	5557790.333 ± 646336.0753	5391568.333 ± 641614.4441
p-JNK	2263950 ± 945888.2826	4457850.667 ± 837962.7502	4854315 ± 873132.7147
JNK	6300119.333 ± 1434555.254	6440248 ± 1148515.122	6535903.667 ± 963618.2061
p-ERK	2427916.667 ± 672377.8107	5157352.222 ± 1753755.579	4376252 ± 2678839.805
ERK	6073408.667 ± 1549168.341	5833759.333 ± 1231988.522	5741769.667 ± 1146250.991
p-p65	3773459.667 ± 1331876.524	6164454.333 ± 2833435.31	3364072.667 ± 871356.6193
p65	4373600 ± 1329434.698	4804302 ± 1449740.573	4646272 ± 2060754.698
kifc2	2555686.333 ± 557289.6244	4625673.333 ± 1546544.859	2459396.667 ± 883194.66
b-actin	5727864 ± 1162924.528	5377267.333 ± 1525717.347	5362642.333 ± 1537171.758
p-p38/p38	0.402343002 ± 0.087260119	0.84783655 ± 0.147494724	0.91421446 ± 0.062387283
p-JNK/JNK	0.35762829 ± 0.112298513	0.694540794 ± 0.069043144	0.753994007 ± 0.166893937
p-ERK/ERK	0.419109933 ± 0.164330999	0.929059016 ± 0.4308272	0.828992306 ± 0.636909459
p-p65/65	0.891696597 ±	1.296254434 ±	0.792232715 ±

	0.304460545	0.544798659	0.322092283
kifc2/b-actin	0.450078261 ± 0.087067156	0.85558785 ± 0.167474615	0.454310359 ± 0.100061009
Relative p-p38/p38	1 ± 0	2.134306312 ± 0.31025367	2.348181766 ± 0.571916748
P value (Relative p-p38/p38)	Control vs. CX3CL1 (0.0236), Control vs. CX3CL1+SB203580 (0.0109), CX3CL1 vs. CX3CL1+SB203580 (0.7738).		
Relative p-JNK/JNK	1 ± 0	2.05798528 ± 0.553774819	2.163289347 ± 0.266022622
P value (Relative p-JNK/JNK)	Control vs. CX3CL1 (0.0248), Control vs. CX3CL1+SB203580 (0.0164), CX3CL1 vs. CX3CL1+SB203580 (0.9305).		
Relative p-ERK/ERK	1 ± 0	2.176335564 ± 0.154773017	1.81138881 ± 0.693956339
P value (Relative p-ERK/ERK)	Control vs. CX3CL1 (0.0294), Control vs. CX3CL1+SB203580 (0.1129), CX3CL1 vs. CX3CL1+SB203580 (0.5544).		
Relative p-p65/65	1 ± 0	1.422229643 ± 0.144437979	0.899236993 ± 0.207705432
P value (Relative p-p65/65)	Control vs. CX3CL1 (0.0283), Control vs. CX3CL1+SB203580 (0.6913), CX3CL1 vs. CX3CL1+SB203580 (0.011)		
Relative kifc2/b-actin	1 ± 0	1.909325746 ± 0.232989427	1.011630091 ± 0.135934991
P value (Relative kifc2/b-actin)	Control vs. CX3CL1 (0.0009), Control vs. CX3CL1+SB203580 (0.9954), CX3CL1 vs. CX3CL1+SB203580 (0.001)		

Figure 5E

	Control	CX3CL1
p-p38	4617583 ± 981924.3924	6967684.667 ± 2208592.725
p38	6488716 ± 1138340.801	6496969.667 ± 1749398.317
p-JNK	3604636.667 ± 941581.7503	7257386.833 ± 977990.2888
JNK	5304206.333 ± 1502667.182	5538910.333 ± 1670438.297
p-ERK	5585853 ± 1404609.934	8531576.167 ± 3039181.008
ERK	7775410.667 ± 2234422.081	7569646.333 ± 2458384.678
b-actin	8206425.667 ± 1704959.55	8232709.667 ± 2000392.571
p-p38/p38	0.716056871 ± 0.128108992	1.068446803 ± 0.108891919
p-JNK/JNK	0.685162739 ± 0.101999177	1.353184068 ± 0.224998203
p-ERK/ERK	0.724833644 ± 0.03585708	1.121539765 ± 0.057582335
Relative p-p38/p38	1 ± 0	1.507568081 ± 0.136573036
Relative p-JNK/JNK	1 ± 0	2.009607099 ± 0.504854608
Relative p-ERK/ERK	1 ± 0	1.549574634 ± 0.106011522

Figure 5F

	Control	CX3CL1
p-p65	4834970 ± 1246004.994	6727826.841 ± 2097340.278
p65	6643056 ± 1887200.398	6742576.333 ± 2373707.938
b-actin	6891550 ± 1030612.805	6580483.667 ± 1151009.924
p-p65/65	0.764022026 ± 0.240445109	1.040816437 ± 0.259855505
Relative p-p65/65	1 ± 0	1.389343996 ± 0.143655463

Figure 4I

	BV2-derived EVs	CX3CL1 BV2-derived EVs	BV2 Lvsate	CX3CL1 BV2 Lvsate
TSG101	2670473.333 ± 523247.0661	4916459.333 ± 1193905.069	1832554.667 ± 350640.5297	2717974.667 ± 703405.2439

CD63	2281888 ± 606341.3869	3518957.333 ± 396486.1508	3308139.667 ± 1189937.344	5253852.333 ± 1214010.259
CALNEXIN	20266.66667 ± 5019.335049	36063 ± 16596.55262	2570389.333 ± 927509.3468	3255352.667 ± 1712344.913

Data are presented as mean ± SD, n = 3 per group.

P-values were determined by one-way ANOVA followed by Tukey's *post hoc* test.
